# Molecular Imaging in Pancreatic Cancer: Current Applications and Future Perspectives

**DOI:** 10.3390/ph19071078

**Published:** 2026-07-13

**Authors:** Yongshun Liu, Kexin Lan, Zhaonan Sun, Wenpeng Huang

**Affiliations:** 1 Department of Medical Imaging, Peking University First Hospital, Beijing 100034, China; 2010301105@stu.pku.edu.cn; 2 Department of Nuclear Medicine, Peking University First Hospital, Beijing 100034, China; lankexinlan@163.com; 3 Department of Radiology, Geriatric Hospital of Nanjing Medical University (Jiangsu Province Official Hospital), Nanjing 210024, China

**Keywords:** pancreatic cancer, molecular imaging, positron-emission tomography, fibroblast activation protein, integrin

## Abstract

Pancreatic cancer ranks among the most lethal malignancies, characterized by a five-year survival rate of approximately 10%. This dismal prognosis is largely attributable to diagnoses occurring at advanced stages and the inherent limitations of conventional imaging modalities in detecting early lesions, identifying metastases, and assessing tumor heterogeneity. Consequently, there is a critical need for non-invasive imaging techniques capable of visualizing pancreatic cancer lesions to enable accurate diagnosis, risk assessment, and the development of personalized treatment strategies. Molecular imaging, which combines highly specific targeted probes with advanced imaging technologies, offers the potential to elucidate disease-associated pathways. This review provides a comprehensive overview of recent advancements in molecular imaging platforms for pancreatic cancer, including positron emission tomography (PET), single-photon emission computed tomography (SPECT), optical molecular imaging, photoacoustic imaging, and molecular MRI. We begin by elucidating the biological rationale for targeting key molecules, including fibroblast activation protein (FAP), integrins, and programmed death ligand 1 (PD-L1). Moreover, we critically evaluate the development and clinical translation of these probes, highlighting their ability to enhance lesion detectability, characterize intratumoral heterogeneity, and guide both targeted therapy and surgical resection. Compared with existing reviews, this work uniquely integrates a comprehensive cross-modality analysis of the latest molecular imaging strategies for pancreatic cancer. Furthermore, we examine prevailing challenges and emerging frontiers in this domain, specifically focusing on multimodal hybrid imaging, artificial intelligence-driven analytics, and integrated theranostic platforms as pivotal strategies to advance precision oncology.

## 1. Introduction

Pancreatic cancer (PC) is an aggressive digestive system tumor with a notably poor prognosis and a 5-year survival rate of approximately 10% [1,2]. Pancreatic ductal adenocarcinoma (PDAC) constitutes over 90% of all pancreatic cancer cases. The incidence of PC is rising globally, making it the third leading cause of cancer-related death in the United States [3,4]. Currently, the diagnosis and management of PC face numerous challenges. Pancreatic cancer is often misdiagnosed or overlooked due to its early symptoms being nonspecific and subtle, including abdominal pain, jaundice, and unintentional weight loss [5]. As a result, the majority of patients are diagnosed at advanced or metastatic stages, with only 15–20% eligible for curative surgical resection at the time of initial presentation [6,7]. Moreover, PDAC exhibits marked intratumoral heterogeneity and demonstrates intrinsic or acquired resistance to chemotherapy and radiotherapy.

Currently, tissue biopsy is the definitive method for diagnosing PC [1]. However, this method is invasive, carries risks of complications, and is difficult to use for early screening and dynamic monitoring of tumor progression. Therefore, imaging examinations play an irreplaceable role in the early screening, diagnostic staging, and monitoring of efficacy in PC [8]. Conventional imaging methods encompass ultrasound, computed tomography (CT), and magnetic resonance imaging (MRI). Their primary benefits include non-invasiveness and comprehensive assessment capabilities, while their current limitations involve inadequate sensitivity for early diagnosis and limited efficacy in detecting micrometastases and assessing treatment response.

Molecular imaging integrates highly specific targeted probes with advanced imaging technologies to elucidate disease-associated pathways, thereby facilitating early diagnosis and precise monitoring of therapeutic response [9]. Fluorine-18-fluorodeoxyglucose (^18^F-FDG), a non-specific tracer, is extensively utilized for visualizing tumor glucose metabolism, making it the predominant technology for cancer staging and evaluating treatment response [10,11]. However, it also has lower detection rates for small early lesions and low-metabolism lesions. Benign conditions like inflammation and infection can cause interference, resulting in false-positive outcomes [12]. Therefore, investigating advanced imaging techniques is essential for enhancing the diagnosis, treatment, and prognosis of PC. These limitations have sparked interest in molecular imaging techniques that use agents targeting specific cancer molecular characteristics. Specific tracers can probe tumor biology beyond glycolysis, potentially enabling more precise diagnoses [13]. Notably, fibroblast activation protein (FAP)-targeted agents, such as [^68^Ga]Ga-FAPI-04, have demonstrated superior tumor-to-background ratios compared to ^18^F-FDG in PDAC. This improved contrast enables the detection of metastatic lesions that remain occult on conventional imaging modalities [14]. Over the past decade, the field has undergone a distinct paradigm shift, evolving from non-specific metabolic imaging toward the development of increasingly sophisticated targeted molecular probes. In light of these advancements, this review principally examines targeted molecular imaging strategies for PC.

**Figure 1:**
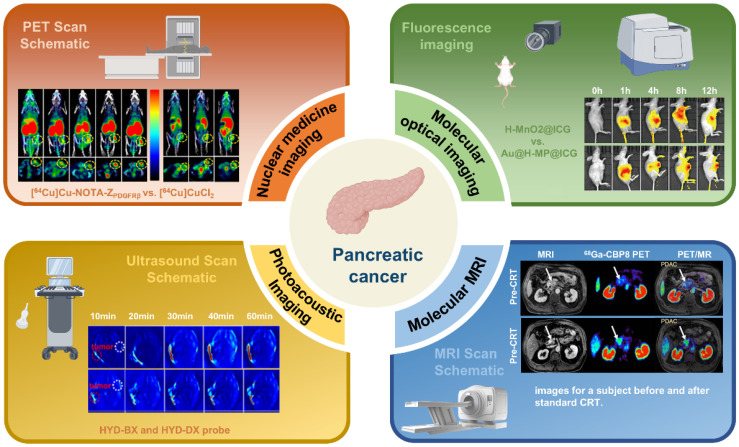
Exploring molecular imaging techniques for pancreatic cancer. This review summarizes recent advances in molecular imaging for pancreatic cancer, covering major technological platforms including nuclear medicine imaging, molecular optical imaging, photoacoustic imaging, and molecular MRI. *PET*, positron-emission tomography; *MRI*, magnetic resonance imaging; *CRT*, chemoradiotherapy. Adapted with permission from refs [15,16,17] (Copyright 2024–2025, American Chemical Society) and ref. [18] (available under a CC-BY 4.0 license, Copyright 2024).

In this review, we first outline the framework for pancreatic cancer imaging diagnosis, define key concepts in molecular imaging, and discuss signaling pathways of relevant biomarkers and therapeutic targets. We have conducted a systematic evaluation of the application value, diagnostic performance, and limitations of targeted molecular imaging technologies such as PET, SPECT, optical molecular imaging, photoacoustic imaging, and molecular MRI over the past five years (Figure 1). Finally, we examine the main challenges impeding clinical translation and highlight new opportunities that could drive future advancements.

### 1.1. Diagnostic Imaging of Pancreatic Cancer

#### 1.1.1. Transabdominal and Endoscopic Ultrasound

Ultrasound (US) is the preferred non-invasive imaging method for pancreatic cancer screening, which has the advantages of simple operation, no radiation, low cost, and repeatability. US enables the delineation of pancreatic morphology, serving as a preliminary screening modality for focal pancreatic lesions. However, the diagnostic utility is significantly operator-dependent and limited by patient body habitus, leading to suboptimal sensitivity for small-volume disease. In the context of PDAC, detection sensitivity is size-stratified: it approaches 95% for tumors exceeding 3 cm, whereas it diminishes to approximately 50% for lesions smaller than 1 cm [19]. Moreover, US technology has difficulty distinguishing PDAC from inflammatory conditions like pancreatitis.

Endoscopic ultrasound (EUS) advances an ultrasound probe through an endoscope into the gastrointestinal tract, enabling close-range scanning of the pancreas. This approach effectively provides significantly higher resolution for pancreatic imaging compared to transabdominal ultrasound. EUS clearly depicts small pancreatic parenchymal lesions and demonstrates superior sensitivity to CT for lesions <2 cm [20,21]. Furthermore, sensitivity is reduced (approximately 80%) for detecting PDAC in patients with concurrent pancreatitis [22]. Moreover, EUS is an invasive procedure with a certain risk of complications (such as bleeding, infection, perforation, etc.), and has high requirements for the technical level of the operating doctor, so it is not suitable for large-scale screening.

In addition, intraoperative ultrasound (IOUS) in PDAC surgery provides critical real-time guidance for lesion localization, resectability assessment, surgical planning, cystic versus solid lesion differentiation, and metastatic surveillance [23,24]. However, IOUS has limitations in identifying superficial and small lesions when compared to visual techniques [25].

#### 1.1.2. Computed Tomography

Computed tomography (CT) is extensively utilized for diagnosing, staging, and planning treatment for pancreatic cancer [26]. It offers high spatial resolution, rapid image acquisition, and clear visualization of lesions and their relationships with adjacent vasculature and organs. CT examinations can be divided into non-contrast and contrast-enhanced types, with contrast-enhanced CT favored for accurate clinical evaluation.

Contrast-enhanced CT enables accurate evaluation of pancreatic tumor invasion extent, involvement of surrounding vessels, adjacent organ infiltration, and presence of distant metastases. This offers crucial data for tumor staging. Multiphase multi-detector row CT (MDCT) demonstrates a sensitivity of 96%, specificity of 33–72%, and positive predictive value (PPV) of 89% in predicting resectability of PDAC [27,28]. Additionally, CT facilitates post-treatment response monitoring by assessing changes in tumor size and metastasis regression. However, limitations include radiation exposure and suboptimal detection rates for subcentimeter lesions (<1 cm) [29]. Contrast administration also carries risks for patients with renal impairment due to its nephrotoxic potential.

#### 1.1.3. Magnetic Resonance Imaging

Magnetic resonance imaging (MRI) is a non-radiative imaging technique that offers high soft tissue resolution and clear visualization of pancreatic parenchyma and duct structures through its multi-sequence and multi-parameter capabilities. MRI demonstrates a sensitivity of 85–93% and a specificity of 72–79% for diagnosing PDAC [30,31].

Magnetic resonance cholangiopancreatography (MRCP) is a non-invasive technique that effectively visualizes pancreatic and bile duct dilation, offering significant diagnostic value for pancreaticobiliary obstructions caused by PC. MRCP demonstrates similar sensitivity to the more invasive ERCP in diagnosing PDAC [32]. However, MRI examination has limitations such as long imaging time, high cost, and inapplicability to patients with metal implants in the body.

## 2. Molecular Imaging Technology in the Detection of Pancreatic Cancer

Molecular imaging employs targeted probes to label biological markers, enabling accurate detection of early disease changes and supporting personalized diagnosis and treatment [33]. Current molecular imaging techniques for PC detection encompass nuclear medicine imaging, optical imaging, molecular MRI, and photoacoustic imaging (PAI).

### 2.1. Nuclear Medicine Imaging

#### 2.1.1. Positron Emission Tomography (PET) Imaging

PET is a functional molecular imaging modality that leverages the distinct metabolic hyperactivity characteristic of malignant tissues. The fundamental principle involves the intravenous administration of a radiolabeled metabolic tracer. Due to their upregulated metabolic rates, neoplastic cells exhibit avid uptake of this substrate. The ensuing annihilation photons are detected by the PET scanner, facilitating precise tumor localization and characterization. Currently, PET represents the most widely used molecular imaging modality for PC in clinical practice. It enables comprehensive whole-body screening in a single session, allowing for the rapid identification of distant metastases across various anatomical sites, including lymph nodes, the liver, and the lungs.

The PET tracer ^18^F-FDG is commonly absorbed by cells with high metabolic activity through glucose transporters and is phosphorylated by hexokinase. This enables the visualization of regions with elevated metabolic activity, making ^18^F-FDG PET a powerful tool for detecting and characterizing malignancies, monitoring neurological disorders, and assessing myocardial viability. For PDAC detection, ^18^F-FDG demonstrates superior diagnostic performance compared to CT, with an average sensitivity of 94% (vs. 82%) and specificity of 90% (vs. 75%) [34]. A UK multicenter trial demonstrated that FDG PET/CT is more accurate than MDCT for diagnosing PDAC, with a sensitivity of 92.7% compared to 88.5% (*p* = 0.010) and a specificity of 75.8% versus 70.6% (*p* = 0.023) [35]. However, ^18^F-FDG is a non-specific metabolic substrate, and benign lesions such as inflammation, infection, and tuberculosis can also take up ^18^F-FDG, leading to false positive results [36].

#### 2.1.2. Single-Photon Emission Computed Tomography (SPECT) Imaging

SPECT is a functional molecular imaging modality that generates 3D tomographic reconstructions by detecting gamma photons emitted from radiolabeled tracers distributed within the body [37]. The procedure entails the intravenous administration of a radionuclide-conjugated targeting agent, such as technetium-99m (^99m^Tc). Upon specific accumulation and binding to tumor cells, the resultant gamma emissions are acquired by an SPECT detector system, thereby facilitating high-resolution visualization and characterization of neoplastic lesions.

In pancreatic cancer imaging, SPECT primarily serves to detect metastatic lesions and evaluate tumor metabolic activity. While SPECT/CT is currently not routinely utilized for primary pancreatic malignancy detection, advances in radiotracer development may expand its future clinical role. Compared to PET, SPECT offers lower equipment costs, reduced radiation exposure, and simplified operational procedures [38]. However, limitations include low spatial resolution, suboptimal detection rates for early-stage small lesions, slow imaging acquisition, and challenges in rapid whole-body scanning. Presently, its clinical application remains less extensive than PET and is primarily supplementary to PET-based assessments.

### 2.2. Optical Molecular Imaging

Optical imaging represents a non-invasive molecular imaging modality that leverages light signals to visualize biological processes. Distinguished by its absence of ionizing radiation, rapid acquisition capabilities, cost-effectiveness, and capacity for real-time visualization, this technology is primarily categorized into two distinct approaches: fluorescence molecular imaging (FMI) and bioluminescence imaging (BLI) [39,40]. Currently, its application is predominantly confined to preclinical research and intraoperative navigation in the context of pancreatic cancer, while its broader clinical translation remains an active area of investigation.

#### 2.2.1. Fluorescence Molecular Imaging

Fluorescence Molecular Imaging (FMI) is an advanced optical technique that employs targeted fluorescent probes to label specific biomolecules, such as tumor-associated markers [41]. Upon excitation by appropriate wavelengths, these probes emit detectable signals, facilitating real-time tumor localization and visualization. FMI is characterized by its high sensitivity, which enables the early detection of lesions and provides intraoperative guidance for the precise delineation of tumor margins and micrometastases, thereby minimizing residual disease [42]. As a non-ionizing modality, FMI allows for serial examinations, making it particularly suitable for longitudinal post-treatment monitoring. Commonly utilized contrast agents include near-infrared (NIR) dyes, quantum dots (QDs), and gold nanoparticles. However, the clinical utility of FMI is constrained by the limited tissue penetration depth of fluorescent signals, which hinders the detection of deep-seated lesions. Furthermore, image quality can be compromised by tissue autofluorescence, which introduces background noise and reduces the signal-to-noise ratio [43,44].

#### 2.2.2. Bioluminescence Imaging

Bioluminescence imaging (BLI) utilizes genetic engineering strategies to stably integrate luciferase reporter genes into tumor cell genomes. Upon expression, the luciferase enzyme catalyzes an oxidative reaction with exogenously administered luciferin substrates, emitting photons that are captured by highly sensitive charge-coupled device (CCD) cameras to facilitate precise tumor visualization [45,46]. BLI is distinguished by its exceptional sensitivity, enabling the detection of minimal residual disease and micrometastatic populations. Furthermore, the technique benefits from a negligible background signal due to the absence of endogenous autofluorescence, thereby yielding superior image contrast. As a non-ionizing modality, BLI permits safe, longitudinal monitoring of tumor dynamics in vivo. However, its clinical translation is currently constrained by the limited penetration depth of visible light through biological tissues, which hinders the detection of deep-seated lesions in large animal models and humans [47]. Consequently, BLI remains predominantly utilized in preclinical pancreatic cancer research, where it serves as a critical tool for the real-time assessment of tumor progression, metastatic spread, and therapeutic efficacy.

### 2.3. Molecular Magnetic Resonance Imaging

Molecular MRI builds upon conventional MRI by integrating molecularly targeted probes to visualize tumors at the molecular level [48]. Its core principle involves targeted probes binding specifically to PC cells. Subsequent MRI detects probe-induced signal changes, enabling precise tumor localization and characterization. Compared to nuclear medicine imaging, MRI inherently possesses lower sensitivity for detecting molecular and cellular events [49]. To mitigate this limitation, probes are often designed to incorporate multiple contrast elements (e.g., nanoparticles) or utilize enzyme-mediated signal amplification mechanisms [50]. However, limitations involve significant challenges in probe development, high costs, biological safety concerns for some probes, and lengthy scan times.

### 2.4. Photoacoustic Imaging

Photoacoustic imaging (PAI) is an emerging hybrid modality that synergistically integrates the high contrast of optical imaging with the deep penetration and spatial resolution of ultrasound [51]. By combining the superior molecular sensitivity of optical methods with the enhanced depth capability of ultrasound, PAI facilitates label-free or targeted molecular imaging without ionizing radiation and supports rapid data acquisition [52]. Consequently, PAI holds significant promise for the early diagnosis and intraoperative navigation of PC [53,54]. Current PAI contrast agents primarily encompass targeted dye-conjugated probes and nanomaterial-based agents, such as gold nanoparticles and carbon quantum dots. These probes can specifically bind to pancreatic cancer cells, enhance photoacoustic signal intensity, and improve imaging sensitivity and specificity. The primary limitations of PAI include its incapacity to image through bone or air-filled structures and the current lack of commercially available intra-operative systems [55].

The diverse array of imaging probes currently under investigation for the molecular imaging of PC demonstrates distinct pharmacokinetic profiles, tumor penetration capacities, and stages of clinical translation (Table 1). Full-length monoclonal antibodies provide superior binding affinity and prolonged tumor retention; however, their slow blood clearance and extended circulation half-lives necessitate delayed imaging windows, typically ranging from 7 to 21 days [56]. Conversely, low-molecular-weight agents, including peptides (e.g., FAPI derivatives, RGD ligands), small molecules, nanobodies, and affibodies, facilitate rapid tissue infiltration and enable high-contrast imaging within hours. Nevertheless, in the context of abdominal imaging, significant renal retention and elevated hepatic background activity remain critical confounding factors. Meanwhile, nanoparticle-based platforms offer capabilities for multimodal imaging and stimuli-responsive theranostics. These systems remain predominantly in the preclinical phase, hindered by challenges related to manufacturing scalability and the need for rigorous long-term biosafety assessment.

## 3. Molecular Mechanisms Underlying Targets and Biomarkers in Pancreatic Cancer

Hereditary and epigenetic factors drive PC development, often progressing from precursor lesions such as pancreatic intraepithelial neoplasia (PanIN) to invasive carcinoma through a multistep process [57]. The disease is driven by a complex network of genetic changes, primarily involving the canonical driver mutations KRAS, CDKN2A, TP53, and SMAD4, along with additional alterations in PTEN, BRAF, PIK3CA, MYC, GNAS, and LKB1, which together define the malignant phenotype. The aggressive growth, therapeutic resistance, and metastatic tendencies of this disease are driven by dysregulation in key signaling pathways such as RAF/MEK/ERK, PI3K/AKT, TGF-β, and Wnt-Notch [58,59]. The biomarker landscape for PC has expanded considerably beyond the traditional reliance on CA19-9. Protein-level markers, including mesothelin, the uPA-uPAR proteolytic axis, IGF-1R, EGFR, Plectin-1, Trop2, Nectin-4, and Claudin18.2, offer opportunities for both refined diagnostics and targeted therapeutic intervention. Circulating microRNA signatures offer potential as non-invasive tools for early disease detection and monitoring. These biomarkers provide potential targets for tumor-specific imaging, where effective molecular agents require high specific-to-nonspecific binding ratios to ensure signal fidelity. Comprehending these molecular mechanisms is crucial for the development of targeted therapies and imaging methods.

### 3.1. Genetic Alterations

#### 3.1.1. KRAS

The Kirsten rat sarcoma viral oncogene homolog (KRAS) encodes a small GTPase that functions as a pivotal molecular switch, transducing extracellular proliferative cues into intracellular signaling cascades [60]. Under physiological conditions, KRAS cycles between an inactive GDP-bound conformation and an active GTP-bound state; this dynamic equilibrium is tightly regulated by guanine nucleotide exchange factors (GEFs), which promote activation, and GTPase-activating proteins (GAPs), which facilitate hydrolysis-driven inactivation. Upon activation, KRAS triggers downstream signaling predominantly through the RAF/MEK/ERK and PI3K/AKT pathways, thereby orchestrating critical cellular processes including proliferation, survival, and motility [61].

Oncogenic mutations lock KRAS in a persistently active configuration, rendering it a potent driver of malignant transformation. In pancreatic cancer, such alterations represent one of the earliest genetic hits during carcinogenesis, detectable in roughly one-quarter of PanIN-1A lesions and over one-third of PanIN-1B precursor abnormalities [62]. The profound dependency of PC cells on mutant KRAS has been substantiated through functional interrogation using RNA interference and CRISPR-based gene editing approaches. KRAS-mutant pancreatic adenocarcinoma cells exhibit markedly greater sensitivity to KRAS ablation compared with their wild-type counterparts [63]. This addiction arises partly because mutant KRAS establishes robust negative feedback loops that dampen upstream receptor tyrosine kinase signaling, thereby precluding rescue by extracellular growth factor stimulation.

#### 3.1.2. PTEN

Phosphatase and tensin homolog (PTEN) serves as a pivotal negative regulator of the PI3K signaling axis. Functioning as a dual-specificity phosphatase, PTEN attenuates phosphoinositide-mediated proliferative and survival signals. Under physiological conditions, PTEN translocates from the cytosol to the plasma membrane, where it catalyzes the dephosphorylation of phosphatidylinositol (3,4,5)-trisphosphate to phosphatidylinositol (4,5)-bisphosphate. This reaction directly antagonizes PI3K activity, thereby restraining aberrant Akt activation. Consequently, the loss of PTEN function, mediated by genetic deletion, somatic mutation, post-translational modification, or epigenetic silencing, leads to the pathological accumulation of phosphatidylinositol (3,4,5)-trisphosphate and subsequent hyperactivation of the PI3K pathway [64].

Experimental evidence derived from conditional knockout models has unequivocally established the tumor-suppressive function of PTEN in pancreatic carcinogenesis. Specifically, the targeted ablation of PTEN within the adult pancreatic ductal epithelium precipitates the formation of IPMNs, a significant proportion of which progress to invasive and metastatic disease. Notably, the invasive components in these models frequently acquire spontaneous KRAS mutations. This observation suggests that PTEN loss synergizes with KRAS activation to drive malignant progression through the convergent RAF/MEK/ERK and PI3K/AKT signaling cascades [65].

#### 3.1.3. BRAF

B-RAF is a serine/threonine kinase within the RAF family that functions immediately downstream of RAS proteins in the MAPK signaling cascade [66]. Although the canonical V600E substitution in B-RAF is markedly less prevalent in pancreatic cancer than in melanoma or colorectal cancer, occurring in approximately 3% of PDAC cases, its presence delineates a molecularly distinct tumor subgroup. Notably, KRAS and B-RAF mutations are mutually exclusive, indicating that either alteration alone is sufficient to drive pathway activation [67,68].

Genetically engineered mouse models have demonstrated B-RAF’s oncogenic potential in the pancreas. Conditional expression of B-RAF V600E under pancreatic lineage-specific promoters elicits near-complete replacement of normal exocrine tissue with PanIN-like precursor lesions. In the same context, the PIK3CA H1047R activating mutation does not induce similar premalignant changes, even after prolonged observation, highlighting the distinct transforming potential of MAPK compared to PI3K pathway activation in pancreatic carcinogenesis [69].

#### 3.1.4. PIK3CA

PIK3CA, which encodes the catalytic p110α subunit of phosphatidylinositol 3-kinase (PI3K), represents one of the most frequently mutated oncogenes in human solid malignancies [70]. In conventional PDAC, PIK3CA mutations are relatively rare, occurring in approximately 3–5% of cases. Conversely, the H1047R hotspot mutation exhibits a markedly higher prevalence in pancreatic intraductal tubular papillary neoplasms (ITPNs). This uncommon premalignant lesion is distinguished from the more prevalent IPMN by distinct histological and molecular profiles [71,72]. Murine models expressing constitutively active PIK3CA rapidly develop PanINs within days, progressing to invasive carcinoma within weeks. These tumors exhibit morphological features reminiscent of Kras-driven lesions and demonstrate concurrent ERK1/2 activation, suggesting critical PI3K/MAPK pathway crosstalk during tumorigenesis [73].

#### 3.1.5. MYC

The MYC family of transcription factors plays a pivotal role in orchestrating cellular metabolism, proliferative potential, and differentiation programs. Under physiological conditions, MYC expression is stringently regulated; however, in a broad spectrum of human malignancies, it becomes aberrantly upregulated through diverse mechanisms, including gene amplification, transcriptional dysregulation, and enhanced protein stability. Notably, MYC amplification is identified in approximately 17% of pancreatic acinar cell carcinomas, a rare and highly aggressive subtype of pancreatic cancer. Furthermore, elevated MYC expression has also been documented in a subset of conventional PDAC cases [74,75,76]. Mechanistic studies demonstrate that MYC cooperates with the peptidyl-prolyl cis-trans isomerase PIN1 to upregulate NRF2 expression, thereby shielding cancer cells from mitochondrial respiratory compromise induced by constitutive KRAS signaling [77].

#### 3.1.6. GNAS

The GNAS locus encodes the Gsα subunit, a pivotal transducer in G-protein-coupled receptor (GPCR) signaling that stimulates adenylyl cyclase to catalyze the production of cyclic AMP (cAMP). Oncogenic mutations affecting critical arginine residues, predominantly R201C and R201H, abolish the intrinsic GTPase activity of Gsα. This loss of function locks the protein in an active GTP-bound state, driving constitutive downstream signaling independent of ligand stimulation [78,79]. Within the landscape of pancreatic neoplasia, GNAS mutations exhibit a distinct lineage-specific distribution. They are notably absent in conventional PDAC but are prevalent in IPMNs, with reported frequencies ranging from 48% to 66% [80,81]. Furthermore, these mutations are detected across the entire histopathological spectrum of IPMNs, from low-grade dysplasia to invasive carcinoma. This ubiquitous presence suggests that GNAS alterations function as early driver events crucial for tumor initiation rather than markers of late-stage progression [80,82].

#### 3.1.7. LKB1/STK11

Liver kinase B1 (LKB1), alternatively designated as serine/threonine-protein kinase 11 (STK11), functions as a pivotal regulator of cellular energy homeostasis and the establishment of cell polarity [83]. Through the phosphorylation-dependent activation of AMP-activated protein kinase (AMPK) family members, LKB1 orchestrates a comprehensive transcriptional and metabolic reprogramming in response to energetic stress. Originally characterized as the tumor suppressor gene underlying Peutz–Jeghers syndrome, LKB1 deficiency is associated with this autosomal dominant disorder, which is clinically defined by the presence of gastrointestinal hamartomatous polyps and a markedly elevated susceptibility to malignancy [84]. Somatic LKB1 alterations are encountered in a subset of IPMNs. Murine models combining conditional Kras G12D activation with LKB1 inactivation in adult pancreatic ducts develop gastric-type IPMNs that faithfully recapitulate key features of the human disease counterpart [85,86]. These observations support a model wherein mutant KRAS provides the initial oncogenic stimulus while LKB1 loss removes an important barrier, permitting clonal expansion and neoplastic progression within the pancreatic ductal system.

#### 3.1.8. SMAD4

SMAD4 (DPC4) is inactivated in approximately 30–50% of PDAC and serves as the central transducer of canonical TGF-β signaling [87,88]. In the context of oncogenic KRAS and mutant TP53, SMAD4 loss paradoxically promotes tumor progression by abrogating cytostatic TGF-β responses while permitting pro-metastatic and immunosuppressive TGF-β effects [88,89]. Recent isogenic models have revealed that SMAD4 deletion profoundly remodels the TME in a KRAS-allele-specific manner. In KRASG12D p53-mutant PDAC, SMAD4 loss attenuates desmoplasia (reduced collagen and αSMA), depletes myofibroblastic CAFs (myCAFs) and antigen-presenting CAFs (apCAFs), and expands inflammatory CAFs (iCAFs) through IL-1α/IL-1β–driven JAK/STAT and NF-κB activation [90,91]. This fibro-inflammatory stroma is further enriched in pro-tumorigenic T3 neutrophils and depleted of NK cells and CD8+ T cells, fostering immune evasion [88]. The biological consequences of SMAD4 loss are further modulated by the specific KRAS mutation present. Although SMAD4 deletion in both KRASG12D and KRASG12V backgrounds increases neutrophil infiltration and alters macrophage composition through TNF and CXCL1/CXCL2 signaling, only KRASG12D SMAD4-deficient tumors exhibit marked upregulation of JAK/STAT signaling and therapeutic vulnerability to JAK inhibitors such as AZD1480. In contrast, KRASG12V SMAD4-deleted PDAC lacks this JAK/STAT dependency, underscoring that oncogenic context dictates the signaling rewiring consequent to SMAD4 inactivation [92]. TGF-β pathway inhibition with galunisertib has demonstrated preclinical efficacy in SMAD4-deficient settings and may warrant exploration in combination with chemotherapy or immunotherapy [93].

### 3.2. MicroRNA Biomarkers

MicroRNAs (miRNAs) are endogenous, non-coding RNA molecules that exert pivotal regulatory control over gene expression at the post-transcriptional level [94]. In PC, miRNA dysregulation is well-documented; distinct miRNA signatures have been characterized in tumor tissues, pancreatic juice, blood, plasma, and other biofluids when compared to normal pancreatic tissue or samples from patients with chronic pancreatitis [95,96]. Notably, specific miRNAs have been identified as critical modulators of the canonical driver mutations associated with PDAC. For instance, the downregulation of miR-193b and miR-143-3p directly upregulates KRAS expression, consequently enhancing cancer cell proliferation in both in vitro and in vivo models [97,98].

The MAPK/ERK and PI3K/AKT signaling cascades are pivotal regulators of PDAC cell proliferation and survival, subject to extensive modulation by miRNAs. Specifically, miR-21 potentiates EGF-induced proliferation in pancreatic cancer cells by repressing Spry2, a negative regulator, thereby resulting in the concomitant activation of both the MAPK/ERK and PI3K/AKT pathways [99]. In contrast, the tumor-suppressive miR-29c directly targets MAPK1, leading to the suppression of ERK/MAPK signaling and a subsequent attenuation of cellular proliferation and invasion [100]. Similarly, the downregulation of miR-98-5p results in the upregulation of MAP4K4, which drives proliferative and metastatic phenotypes via MAPK/ERK pathway activation [101]. Furthermore, the miR-221/222 cluster promotes oncogenic progression by dysregulating matrix metalloproteinases (MMPs) through the direct inhibition of tissue inhibitor of metalloproteinases 2 (TIMP-2) [102]. Regarding the PI3K/AKT axis, miR-194-5p enhances cancer cell proliferation and migration by targeting SOCS2, an inhibitor that normally constrains this pathway; its repression thus leads to PI3K/AKT hyperactivation [103]. Conversely, miR-30d exerts anti-proliferative and anti-invasive effects by suppressing the SOX4-mediated activation of the PI3K/AKT signaling cascade [104].

Epithelial–Mesenchymal Transition (EMT) is essential for PDAC cells to develop invasive and metastatic characteristics. The miR-200 family and miR-205 are crucial for preserving epithelial integrity and inhibiting EMT by downregulating ZEB1 and ZEB2 transcription factors, which maintains E-cadherin expression [105,106]. miR-361-3p promotes EMT by targeting dual-specificity phosphatase 2 (DUSP2), resulting in ERK1/2 pathway activation and enhanced liver metastasis [107]. Additionally, miR-24-3p contributes to PDAC aggressiveness by triggering EMT through targeting LAMB3 and ASF1B [108,109]. Hypoxic conditions within the pancreatic tumor microenvironment further drive EMT progression. miR-301a facilitates mesenchymal transition by directly targeting TP63 and increasing the expression of hypoxia-inducible factor 1-alpha (HIF-1α) [110]. In hypoxia, pancreatic cancer cells secrete exosomal miR-30b-5p, enhancing angiogenesis by suppressing gap junction protein alpha 1 (GJA1) in endothelial cells [111]. Macrophage-derived exosomal miR-501-3p promotes cancer cell migration, invasion, and metastasis by targeting TGF-β receptor III (TGFBR3), thereby activating TGF-β signaling [112].

A hallmark of PDAC is metabolic reprogramming towards aerobic glycolysis (the Warburg effect). miR-3662 reverses gemcitabine resistance and suppresses glycolysis by directly targeting HIF-1α and reducing expression of glycolytic genes [113]. miR-124 suppresses the progression of PDAC by directly targeting monocarboxylate transporter 1 (MCT1), a critical downstream effector of HIF-1α. The downregulation of MCT1 disrupts intracellular pH homeostasis, thereby attenuating tumor cell proliferation and invasive capacity [114]. miR-323a-3p targets hexokinase 2 (HK-2), thereby inhibiting cancer cell glycolysis and proliferation [115]. KRAS activation has been shown to ablate miR-29 expression, leading to increased extracellular matrix deposition by cancer-associated fibroblasts (CAFs) and promoting cancer cell colony formation [116].

### 3.3. Protein Biomarkers

#### 3.3.1. Mesothelin

Mesothelin is a glycosylphosphatidylinositol-anchored membrane protein typically found in the mesothelial cells of the pleura, peritoneum, and pericardium [117]. Experimental attenuation of mesothelin expression through RNA interference significantly suppresses the proliferative capacity of PC cells, establishing this protein as a functionally relevant driver of PDAC pathophysiology and a rational target for therapeutic intervention. Gene expression profiling studies originally identified mesothelin as a differentially expressed marker of pancreatic adenocarcinoma, and subsequent analyses using in situ hybridization and reverse transcription PCR have confirmed elevated mesothelin messenger RNA levels in both resected tumor specimens and established cancer cell lines [118]. These findings have motivated the development of mesothelin-directed therapeutics, including immunotoxin conjugates and antibody–drug conjugates, some of which have entered clinical evaluation for advanced pancreatic cancer.

#### 3.3.2. uPA/uPAR

The urokinase plasminogen activator (uPA) and its receptor (uPAR) are crucial in PDAC pathophysiology, facilitating tumor invasion, migration, and metastasis via coordinated proteolytic and non-proteolytic processes [119,120].

Plasmin, the broad-spectrum serine protease central to uPA/uPAR activity, mediates direct degradation of ECM components and activates multiple downstream matrix metalloproteinases (MMPs), further contributing to ECM remodeling and stromal degradation [121]. Plasmin also activates latent growth factors such as vascular endothelial growth factor (VEGF), hepatocyte growth factor (HGF), insulin-like growth factor (IGF), and tumor necrosis factor-alpha (TNF-α), which in turn promote tumor cell proliferation, angiogenesis, and lymphangiogenesis [122]. Plasmin bound to the cell surface is shielded from α2-antiplasmin inactivation, maintaining its proteolytic function at the invasive front [123].

Beyond its proteolytic functions, uPAR orchestrates complex intracellular signaling through interactions with vitronectin and transmembrane integrins, activating downstream MAPK, ERK, and JNK pathways that modulate tumor cell adhesion, proliferation, and migration [122]. uPAR-mediated signaling also inhibits the p38 pathway, which regulates apoptosis and G0/G1 cycle arrest, thereby promoting cancer cell survival [124].

#### 3.3.3. IGF-1R

The Insulin-like Growth Factor-1 Receptor (IGF-1R), a transmembrane receptor tyrosine kinase, plays a pivotal role in the pathogenesis and progression of PDAC. In patients with PDAC, elevated intratumoral IGF-1R expression is consistently correlated with higher histological grade and inferior survival outcomes [125,126]. Furthermore, increased concentrations of IGF-1 and its binding proteins (IGFBPs) have been documented in both the serum and tumor tissues of affected individuals [127]. In vitro studies demonstrate that IGF-1 stimulates the proliferation of PDAC cell lines, an effect that can be abrogated by IGF-1R-specific monoclonal antibodies [128]. Moreover, IGF-1R signaling confers resistance to apoptosis while promoting cellular proliferation and motility, thereby underscoring its critical mitogenic and pro-metastatic functions [129,130].

A pivotal crosstalk between IGF-1R signaling and the oncogenic driver KRAS has been firmly established in PDAC. KRAS mutations are prevalent in the vast majority of PDAC cases and constitute one of the earliest oncogenic events sufficient to initiate pancreatic tumorigenesis [131]. In pancreatic ductal epithelial cells, the activation of PI3K signaling and subsequent proliferation are contingent upon mutated KRAS, downstream MAPK pathway engagement, and autocrine IGF-1R activation mediated by the IGF-2 ligand [132]. Notably, epithelial cells harboring mutant KRAS remain dependent on functional IGF-1R for malignant progression; indeed, only the concomitant inhibition of both IGF-1R and MAPK signaling significantly compromises the viability of these cells [132]. Furthermore, active AKT upregulates IGF-1R expression in PDAC cell lines, whereas suppression of AKT signaling downregulates IGF-1R levels. This reciprocal regulation establishes a positive feedback loop that potentiates cancer cell invasiveness [133].

#### 3.3.4. EGFR

The epidermal growth factor receptor (EGFR), a transmembrane tyrosine kinase, is overexpressed in 30% to 89% of PDAC. Upon activation, EGFR initiates multiple downstream oncogenic signaling cascades—including the RAS/RAF/MEK/ERK, PI3K/AKT/mTOR, and JAK/STAT pathways—thereby driving angiogenesis, conferring resistance to apoptosis, and fostering therapeutic resistance [134]. Elevated EGFR expression serves as an independent prognostic indicator of poor clinical outcomes, correlating significantly with lymph node metastasis and diminished overall survival. Consequently, combinatorial strategies integrating EGFR-targeted agents with standard chemotherapy have been extensively investigated to enhance therapeutic efficacy in advanced PDAC [135,136].

#### 3.3.5. Plectin-1

Plectin-1, a cytolinker organizing cytoskeletal architecture, is aberrantly exposed on the exterior surface of pancreatic ductal carcinoma cells while remaining sequestered in the cytoplasm of healthy pancreatic tissue [137,138]. It distinguishes PDAC from chronic pancreatitis, with 60% of PanIN III lesions positive, compared with 0–3.85% of PanIN I/II. All assessed metastatic foci express plectin-1. As the first biomarker enabling in vivo discrimination of primary and metastatic PDAC via tPTP imaging, it exhibits 1.9–2.9-fold higher retention in tumors than normal tissue, supporting early detection and image-guided intervention [139].

#### 3.3.6. Trop2

Trophoblast cell surface antigen 2 (Trop2) is significantly overexpressed in pancreatic cancer, with its high expression levels associated with tumor occurrence, invasiveness, and unfavorable prognosis, especially in poorly differentiated tumors [140,141]. Trop2 drives pancreatic cancer progression through multiple interconnected signaling pathways. It activates the MAPK/ERK cascade, leading to AP-1 transcription factor induction, p27 downregulation, and subsequent cyclin D1/E-CDK complex formation that accelerates cell cycle progression and proliferation [142,143]. Trop2 also triggers the PI3K/AKT signaling axis, thereby enhancing tumor cell migration, invasion, and metastatic dissemination [144,145]. Through these coordinated molecular networks, Trop2 establishes itself as a central oncogenic driver and promising therapeutic target in pancreatic cancer.

#### 3.3.7. Nectin-4

Nectin-4, alternatively designated as poliovirus receptor-related protein 4 (PVRL4), is a calcium-independent cell adhesion molecule belonging to the immunoglobulin superfamily. Nectin-4 expression exhibits a significant positive correlation with increased tumor burden, enhanced proliferative capacity, and robust intratumoral angiogenesis, collectively serving as indicators of poor prognosis in patients with PDAC [146,147]. The oncogenic potency of Nectin-4 is primarily mediated through the activation of the PI3K/AKT signaling cascade. Mechanistically, Nectin-4 forms a functional complex with afadin, an F-actin-binding protein, to stabilize intercellular junctions; this interaction subsequently triggers PI3K/AKT pathway activation, thereby fostering tumor cell survival and conferring resistance to apoptosis [148]. Furthermore, Nectin-4 contributes to tumorigenesis via the modulation of the JAK-STAT signaling axis [149]. At the immunological level, Nectin-4 engages the inhibitory checkpoint receptor TIGIT, resulting in the suppression of natural killer (NK) cell cytotoxicity. This interaction facilitates immune evasion and accelerates disease progression [150].

#### 3.3.8. Claudin 18.2

Claudin 18.2 (CLDN18.2), a tight junction component normally restricted to differentiated gastric epithelium, undergoes ectopic upregulation in pancreatic malignancies, where it serves as both a facilitator of neoplastic transformation and a clinically actionable target [151,152,153]. Intriguingly, robust CLDN18.2 positivity in pancreatic neoplasms tends to parallel well-differentiated histology and is linked to more favorable survival trajectories relative to low-expressing counterparts, distinguishing it from many other oncologic markers [152].

The molecular circuitry governing CLDN18.2 induction in pancreatic cancer cells is multifaceted. At the transcriptional level, protein kinase C (PKC) operates as a key activator: upon stimulation, PKC triggers AP-1 phosphorylation, enabling this transcription factor to dock onto cAMP-responsive motifs within the CLDN18.2 promoter and drive gene expression [154]. Epigenetic constraints also modulate this axis; dense methylation of CpG islands within the CLDN18.2 promoter region imposes transcriptional silencing, and demethylating interventions can reverse this suppression in pancreatic cancer models [155,156]. Collectively, these attributes—namely, its early emergence in pancreatic carcinogenesis, robust expression in metastatic lesions, and accessible extracellular localization—position CLDN18.2 as a compelling target for both molecular diagnostics and antibody-based therapeutics [157,158].

## 4. Molecular Imaging of Pancreatic Cancer

Significant progress has been made in targeted molecular imaging of pancreatic cancer in the last five years, marked by the creation of various radiolabeled probes aimed at specific tumor-associated molecular markers (Table 2). These probes enable non-invasive visualization of tumor biology beyond glucose metabolism, offering potential improvements in lesion detection, patient stratification, treatment response monitoring, and theranostic management. This section systematically reviews recent advancements in targeted molecular imaging probes for pancreatic cancer, categorized by biological targets.

To systematically evaluate the current landscape of targeted molecular imaging in pancreatic cancer, we conducted a comprehensive literature search of PubMed/MEDLINE, Embase, and the Cochrane Library through June 2026. The search used the following structured Boolean query combining four concept blocks: (A) ‘Pancreatic’ OR ‘Pancreas’; (B) ‘Cancer’ OR ‘Carcinoma’ OR ‘Tumor’ OR ‘Tumor’ OR ‘Neoplasm’; (C) ‘Molecular imaging’ OR ‘ImmunoPET’ OR ‘PET’ OR ‘PET/CT’ OR ‘SPECT’ OR ‘Optical imaging’ OR ‘Photoacoustic imaging’ OR ‘Fluorescence imaging’ OR ‘Molecular MRI’ OR ‘Theranostics’; (D) ‘FAP’ OR ‘Fibroblast activation protein’ OR ‘Integrin’ OR ‘αvβ6’ OR ‘αvβ3’ OR ‘PD-L1’ OR ‘Programmed death-ligand 1’ OR ‘Trop2’ OR ‘EGFR’ OR ‘c-Met’ OR ‘CEACAM5’ OR ‘Claudin 18.2’ OR ‘CA19-9’ OR ‘MUC1’ OR ‘MUC5AC’ OR ‘CD47’ OR ‘uPAR’ OR ‘Hsp90’ OR ‘PARP’ OR ‘CSPG4’ OR ‘Biomarker’ OR ‘Molecular target’. The search was restricted to English-language articles published between 2019 and 2026, with supplementary inclusion of seminal pre-2019 works where necessary for contextual background. Reference lists of retrieved articles were manually screened to identify additional relevant publications. The selected studies must meet the following criteria: (i) peer-reviewed publications in journals indexed in the Science Citation Index; (ii) rigorous methodological design with validated research outcomes; (iii) priority given to high-impact original research articles, phase I/II clinical trials, and recent comprehensive reviews. Exclusion criteria included the following: (i) non-peer-reviewed sources (preprints, conference abstracts without full publication); (ii) studies with inaccessible full text where authors could not be contacted; (iii) studies limited to non-pancreatic malignancies without relevance to pancreatic cancer molecular imaging. As this is a narrative review, representative studies are included rather than all available evidence, with priority assigned based on clinical translational relevance, methodological rigor, and novelty of findings.

### 4.1. Targeting Tumor Stroma and Cancer-Associated Fibroblasts

#### 4.1.1. FAP

Fibroblast activation protein (FAP) is a transmembrane serine protease predominantly found on CAFs in over 90% of epithelial tumors, while its expression in normal tissues is minimal. This makes FAP an ideal target for prostate cancer imaging and therapy. A series of FAP inhibitors (FAPIs) labeled with various radionuclides has been developed and extensively evaluated.

[^18^F]F-AlF-NOTA-FAPI-04 has shown high specificity for PC in a study involving 103 treatment-naive patients. PC exhibited significantly higher SUVmax, SUVmean, and total lesion FAP (TLF) expression compared to non-pancreatic tumors (all *p* < 0.001). Tumors located in the pancreatic head or neck showed higher uptake compared to those in the body or tail, with TLF positively correlating with tumor size and stage [159]. [^18^F]FAPI-04 PET/CT was assessed for evaluating pathological response after systemic treatment in a prospective study involving 59 patients. While baseline FAPI uptake parameters did not predict pathologic response, treatment-induced changes in FAPI uptake were significantly associated with treatment response, suggesting that serial FAPI PET imaging may be valuable for selecting candidates for conversion surgery [160].

Numerous studies have evaluated FAPI PET in comparison to conventional [^18^F]FDG PET. [^68^Ga]Ga-FAPI-04 showed a notably higher normalized net activity concentration and target signal than [^18^F]FDG, enabling FAPI PET to identify more tumor lesions in PC [161]. Zhang et al. [163] conducted a prospective comparison of [^68^Ga]Ga-DOTA-FAPI-04 PET/MR and [^18^F]FDG PET/CT with contrast-enhanced CT for preoperative evaluation in 31 patients with pancreatic cancer. FAPI PET/MR achieved N and M staging accuracy of 82.4% and 100%, respectively, versus 58.8% and 90.3% for the conventional approach, and detected more liver metastases with clearer visualization of peritoneal metastases. McGahan et al. [162] reported that [^68^Ga]Ga-FAPI PET/CT prevented futile surgery in 5 of 16 patients with resectable PDAC by detecting CT-occult metastases, and primary tumor SUVmax at 60 min correlated with lymph node ratio (*p* < 0.05).

**Figure 2:**
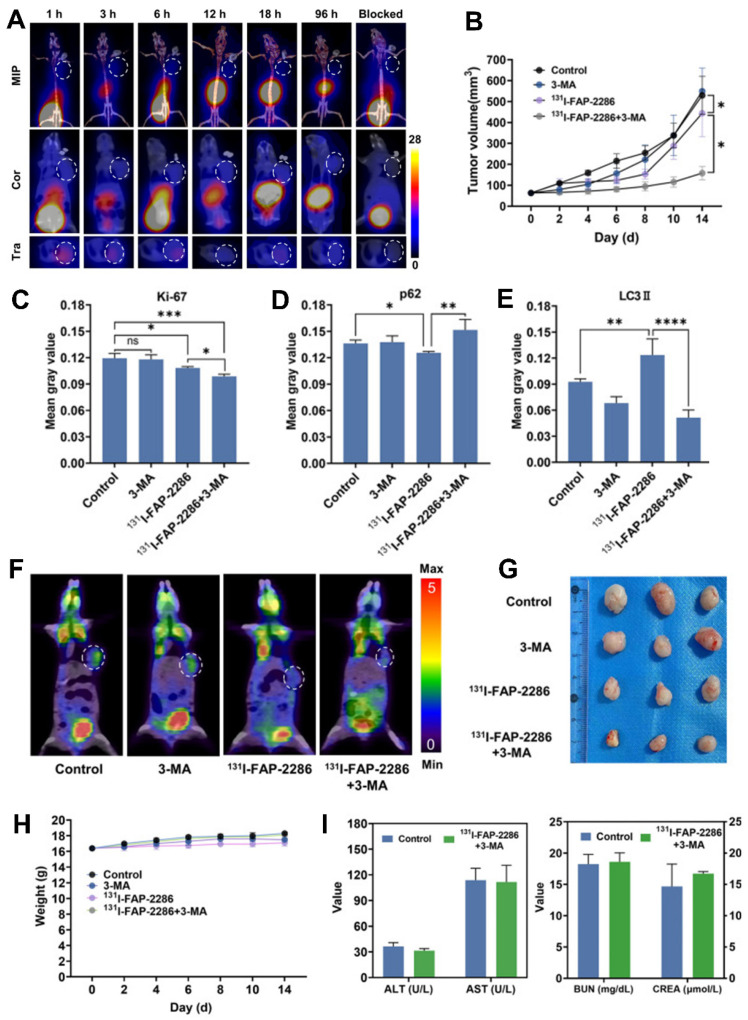
^131^I-FAP-2286 in pancreatic cancer xenografts. (**A**) Representative SPECT/CT images at different time points. (**B**) Tumor growth curves per treatment group. (**C**–**E**) Quantification of Ki-67, p62, and LC3II expression. ns, not significant; * *p* < 0.05, ** *p* < 0.01, *** *p* < 0.001, **** *p* < 0.0001. (**F**) Representative ^18^F-FDG PET/CT images after different treatments. (**G**) Photograph of excised tumors. (**H**) Body weight changes over time. (**I**) Serum ALT, AST, BUN, and CREA levels at 2 weeks post-injection (control vs. ^131^I-FAP-2286+3-MA). The dotted circle points the location of the tumor. Reproduced from ref. [164]. Available under a CC-BY 4.0 license.

Beyond diagnosis, FAP-targeted radioligand therapy has emerged as a promising theranostics strategy. Liu et al. [164] demonstrate that the radiolabeled peptide probe ^131^I-FAP-2286 facilitates specific molecular imaging of CAFs within PDAC via SPECT/CT. Notably, a high tumor-to-background ratio (TBR) was observed exclusively in xenograft models co-implanted with CAFs (Figure 2A). Furthermore, their findings reveal that targeted radionuclide therapy (TRT) employing ^131^I-FAP-2286 induces protective autophagy in PANC-1 tumors, as evidenced by decreased p62 levels, upregulated LC3-II and Ki-67 expression (Figure 2C,D); this adaptive response consequently constrains therapeutic efficacy. Critically, the concomitant administration of TRT and the autophagy inhibitor 3-methyladenine (3-MA) yielded a synergistic antitumor effect, characterized by minimal ^18^F-FDG uptake and maximal tumor growth suppression (Figure 2B,F,G). Importantly, this combinatorial strategy did not exacerbate systemic toxicity, as corroborated by stable body weight, preserved hepatorenal function, and intact histomorphology of major organs (Figure 2H,I). These findings support FAP-targeted peptide imaging and therapy coupled with autophagy modulation as a promising translational approach for stroma-rich PDAC.

#### 4.1.2. TGFβ

**Figure 3:**
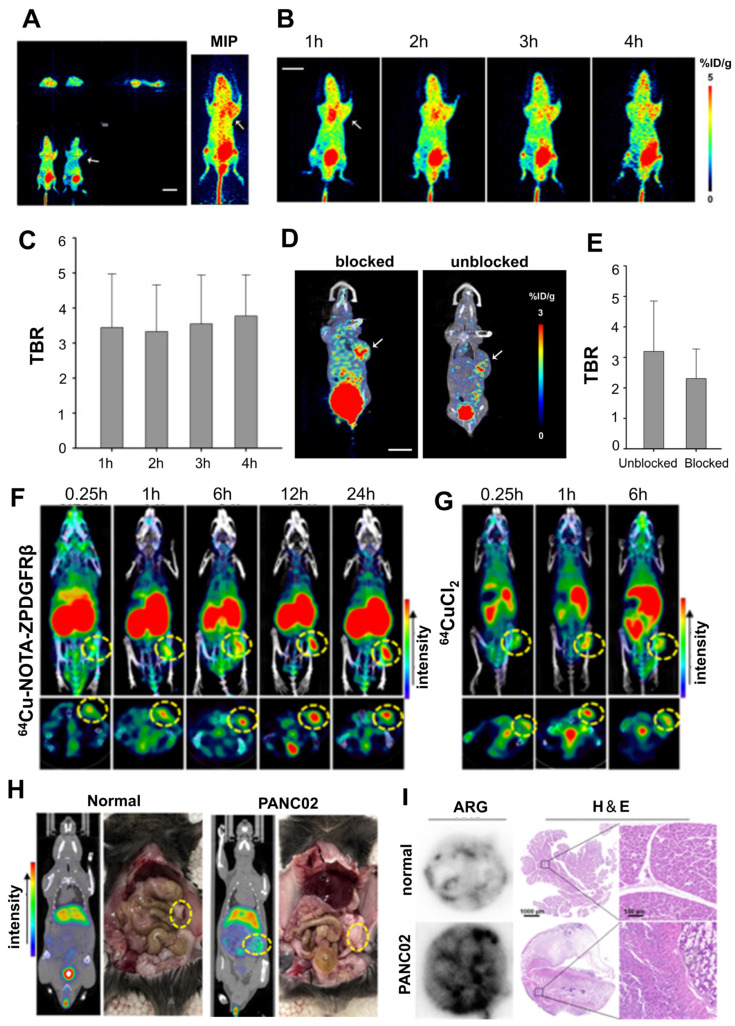
CAF-associated targets in pancreatic cancer xenografts. (**A**) MicroPET images of [^68^Ga]Ga-P144 in PANC-1 tumor-bearing mice at 3 h post-injection (sections and MIP). (**B**) PET images at different time points post-injection. (**C**) Biodistribution in major organs and tumor at different time points (*n* = 5). (**D**) PET/CT images of [^68^Ga]Ga-P144 alone (unblocked) or with blockade (blocked) by excess unlabeled DOTA-P144 at 1 h in the PANC-1 model. (**E**) Tumor-to-muscle ratios (TBR) at 1 h (*n* = 4). Reproduced from ref. [165]. Available under a CC-BY license. (**F**,**G**) Dynamic PET/CT of [^64^Cu]Cu-NOTA-ZPDGFRβ and [^64^Cu]Cu^2+^ in mice bearing PANC02 grafts. (**H**) In situ PET/CT of [^64^Cu]Cu-NOTA-ZPDGFRβ in PANC02 grafts. (**I**) H&E staining and autoradiography of [^64^Cu]Cu-NOTA-ZPDGFRβ in normal pancreas and PANC02 grafts. Reproduced from ref. [16], Copyright 2025, American Chemical Society.

Transforming growth factor-β (TGFβ) is the core upstream target for CAF activation, maintenance, and promotion of tumor function. Li et al. [165] developed and validated a novel TGFβ-targeted PET radiotracer, [^68^Ga]Ga-P144, derived from P144, a high-affinity peptide mimetic of the TGFβ receptor III (TGFβR3) ectodomain. In a PANC-1 pancreatic ductal adenocarcinoma xenograft model, the probe demonstrated rapid and specific tumor accumulation, reaching 6.023 ± 1.370%ID/g at 4 h post-injection. This uptake yielded favorable tumor-to-background contrast, with a tumor-to-muscle ratio of 3.770. High target specificity was corroborated by a significant reduction in tumor uptake following pre-administration of unlabeled (cold) P144 (Figure 3A–E). Immunohistochemical analysis confirmed elevated TGFβ expression in PANC-1 tumors, whereas normal hepatic tissue exhibited negligible expression, thereby validating the biological relevance of the observed imaging signal. Collectively, these findings support [^68^Ga]Ga-P144 as a promising agent for the noninvasive, quantitative visualization of TGFβ activity in malignancies characterized by abundant TGFβ signaling and dense stromal architecture.

#### 4.1.3. PDGFRβ

Platelet-derived growth factor receptor-β (PDGFRβ) represents another CAF-associated target. Li et al. [16] engineered [^64^Cu]Cu-NOTA-ZPDGFRβ, a high-affinity affibody-based PET radiotracer designed to target PDGFRβ expressed on CAFs within PDAC. In vivo evaluations demonstrated rapid tumor accumulation, peaking at 6 h post-injection (7.28 ± 0.92%ID/g), and yielded exceptional imaging contrast. Notably, the tracer achieved a tumor-to-pancreas ratio of 25.9 ± 8.18 at 24 h, facilitating the clear delineation of both subcutaneous (Figure 3F) and orthotopic pancreatic tumors (Figure 3H). In stark contrast, the control group administered with free ^64^Cu^2+^ exhibited only nonspecific background distribution, failing to resolve either tumor model (Figure 3G). Furthermore, ex vivo autoradiography (Figure 3I) corroborated stromal-specific retention, revealing an intense radiotracer signal localized exclusively within the tumor microenvironment, with negligible uptake in adjacent normal pancreatic parenchyma. These findings provide histological-level validation of target engagement and confirm the probe’s high specificity.

#### 4.1.4. Collagen Type I

**Figure 4:**
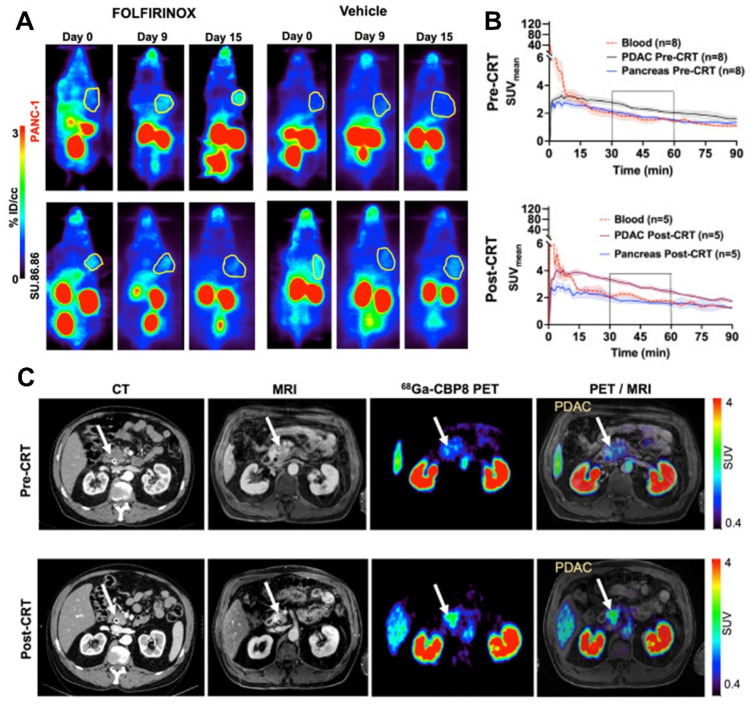
^68^Ga-CBP8 PET captures treatment-induced collagen remodeling in PDAC models and patients. (**A**) Quantitative tumor uptake of ^68^Ga-CBP8 at days 0, 9, and 15 following FOLFIRINOX or vehicle treatment. Tumors are shown in yellow circle. (**B**) Dynamic PET time–activity curves (0–90 min) showing rapid blood clearance, with blood SUVmean approaching uninvolved pancreas SUVmean by 15–30 min. (**C**) Representative axial images before and after CRT: contrast-enhanced CT (late arterial phase), gadolinium-enhanced MRI (portal venous phase), ^68^Ga-CBP8 PET (30–45 min), and fused PET/T1-weighted MR. PDAC tumor is shown with a white arrow. *CRT*, chemoradiotherapy. Reproduced from ref. [18]. Available under a CC-BY 4.0 license.

Type I collagen is a major component of the desmoplastic stroma in PDAC and increases in response to neoadjuvant chemoradiotherapy (CRT), making it an attractive target for monitoring treatment response. In a landmark first-in-human study, Esfahani et al. [18] demonstrated that the type I collagen-targeted PET probe ^68^Ga-CBP8 enables noninvasive, quantitative monitoring of therapy-induced stromal remodeling in PDAC. In preclinical models, probe uptake increased significantly only in FOLFIRINOX-sensitive tumors, mirroring histologically confirmed collagen deposition, while remaining unchanged in resistant tumors (Figure 4A). Critically, in 8 PDAC patients undergoing neoadjuvant CRT, tumor ^68^Ga-CBP8 SUVmean rose significantly from baseline to post-CRT (2.25 ± 0.41 to 2.83 ± 0.30, *p* = 0.01), correlating strongly with collagen proportion area (CPA) measured on resected specimens (Figure 4B). Representative clinical PET/MRI fusion images vividly illustrate this treatment-associated collagen accumulation within the tumor (Figure 4C), establishing collagen PET as a promising functional biomarker of stromal response beyond conventional anatomic imaging.

#### 4.1.5. Fibronectin

Pancreatic stellate cells in the tumor microenvironment secrete extradomain B fibronectin (EDB-FN), which enhances chemoresistance by impeding drug perfusion. Zhang et al. [166] introduced ZD2-Gd-DOTA-Cy7, a dual-modality imaging probe designed to target EDB-FN for near-infrared FMI and MRI applications. In vitro, the concentration of ZD2-Gd-DOTA-Cy7 exhibited a linear relationship with both fluorescence intensity and T1 relaxation time. At 30 min post-injection with a Gd dosage of 0.05 mmol/kg, ZD2-Gd-DOTA-Cy7 achieved 1.44-fold and 1.90-fold greater contrast enhancement compared to free Cy7 and Gd-DOTA, respectively. In the control group, the T1 reduction ratio and normalized tumor background ratio in fibrotic tumor areas were significantly higher by 1.99-fold and 1.78-fold, respectively, compared to the AG-treated group during albumin-bound paclitaxel and gemcitabine chemotherapy monitoring. The combination of fibronectin targeting with multimodal imaging capabilities offers a complementary approach to FAP imaging for comprehensive stromal assessment.

### 4.2. Targeting Integrins

Integrins are heterodimeric transmembrane receptors that mediate cell adhesion, migration, and invasion. Multiple integrin subtypes are overexpressed in PC and have been targeted for molecular imaging. This section covers integrin αvβ6, α6, α5, and bispecific approaches.

#### 4.2.1. Integrin αvβ6

Integrin αvβ6 is an epithelium-specific integrin that is undetectable in healthy adult pancreas but is upregulated in PC, with nearly uniform high expression among patient samples screened. Several PET probes targeting integrin αvβ6 have been developed and evaluated.

**Figure 5:**
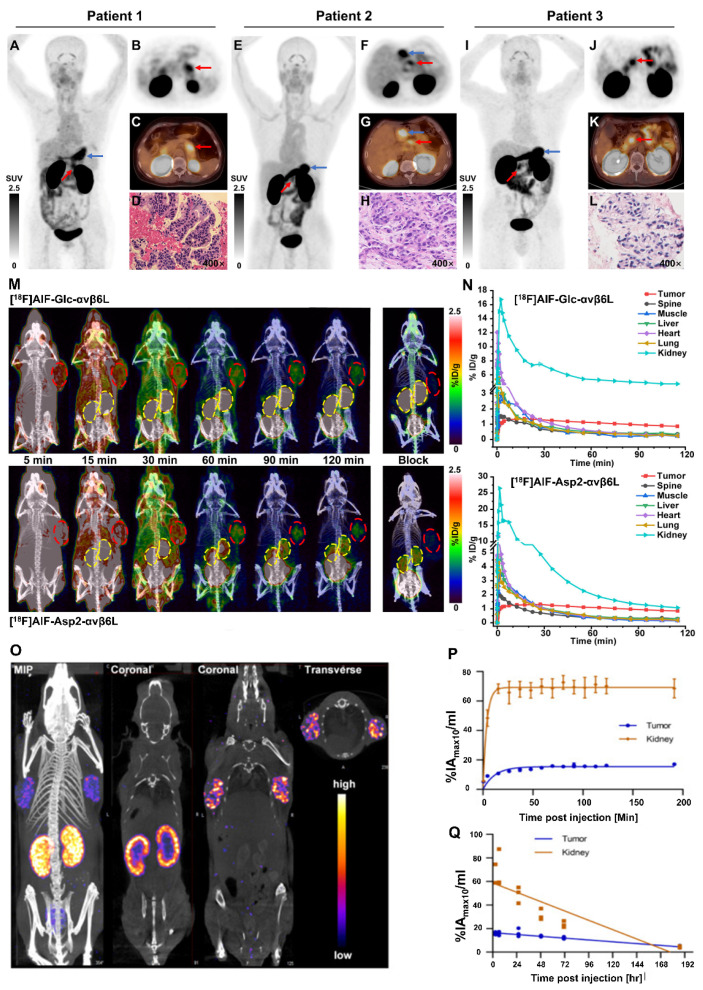
Integrin αvβ6-targeted tracers for pancreatic cancer imaging. [^68^Ga]Ga-αvβ6-2 targeted imaging (MIP, PET, fused images), and H&E staining (400×) of Patient 1 (**A**–**D**), Patient 2 (**E–H**), and Patient 3 (**I**–**L**). Red arrows, pancreatic lesions; blue arrow, diffuse gastric uptake. Reproduced from ref. [168], Copyright 2025, American Chemical Society. (**M**) Dynamic PET/CT of [^18^F]AlF-Glc-αvβ6L and [^18^F]AlF-Asp2-αvβ6L in Capan-2 tumor-bearing mice. Capan-2 tumors are delineated in red dashed circles, and kidneys are delineated in yellow dashed circles. (**N**) Time–activity curves over 2 h post-injection. Reproduced from ref. [167], Copyright 2025, American Chemical Society. (**O**) SPECT/CT of ^177^Lu-DOTA-integrin αvβ6 knottin in Capan-2 xenografts (MIP, coronal, transverse) at 22 h post-injection. (**P**) Early SPECT kinetics in tumor and kidney. (**Q**) Time–activity curve (2–187 h) showing faster renal than tumor clearance. Reproduced from ref. [171]. Available under a CC-BY license. Copyright 2021.

Pang et al. [168] designed [^68^Ga]Ga-αvβ6-2, a PET probe targeting integrin αvβ6, utilizing the dimeric HK peptide sequence. In vitro studies showed that [^68^Ga]Ga-αvβ6-2 had superior binding affinity, cellular uptake, and internalization compared to the monomeric [^68^Ga]Ga-αvβ6-1. Biodistribution and micro-PET/CT imaging studies demonstrated the enhanced imaging capabilities of the dimeric probe. In a first-in-human evaluation of three patients with pancreatic cancer, [^68^Ga]Ga-αvβ6-2 successfully visualized PC lesions, with SUVmax values of 5.09, 4.10, and 5.34 (Figure 5A–L), confirming its translational potential for αvβ6-positive pancreatic cancer detection.

Zhang et al. [167] engineered two novel integrin αvβ6-targeted PET radiotracers, [^18^F]AlF-Glc-αvβ6L and [^18^F]AlF-Asp2-αvβ6L. In Capan-2 PDAC xenograft models, both agents exhibited favorable pharmacokinetic profiles and high target specificity (Figure 5M,N). Notably, [^18^F]AlF-Asp2-αvβ6L demonstrated superior in vivo stability, accelerated renal clearance, and significantly reduced renal retention while maintaining tumor uptake comparable to its counterpart. These attributes position [^18^F]AlF-Asp2-αvβ6L as a promising candidate for the non-invasive PET imaging of PDAC.

Nakamoto et al. [170] conducted a phase I pilot study of ^18^F-FP-R01-MG-F2, a cystine-knot peptide-based αvβ6 probe, in 15 patients with PC. The probe was well tolerated with no adverse events detected, all 15 primary pancreatic tumors along with lung, liver, and peritoneal metastases, though its sensitivity for subcentimeter peripancreatic lymph node metastases was limited.

The theranostic potential of integrin αvβ6 targeting has also been explored. Ganguly et al. [169] developed ^68^Ga-DOTA-5G PET imaging and ^177^Lu-DOTA-ABM-5G (incorporating an albumin-binding moiety) for radioligand therapy.

PET imaging with ^68^Ga-DOTA-5G successfully visualized BxPC-3 pancreatic tumors, while ^177^Lu-DOTA-ABM-5G demonstrated significantly enhanced tumor retention and prolonged median survival from 56 days to 82 or 113 days in mouse models. Sachindra et al. [171] assessed ^177^Lu-DOTA-integrin αvβ6 cystine knot peptide in Capan-2 xenografts, showing strong affinity and specificity for integrin αvβ6-overexpressing PDAC cell lines, including BxPC-3 and Capan-2. Notably, off-target expression was negligible in murine hepatic and renal tissues at the mRNA level. In Capan-2 xenograft models, the probe demonstrated significant tumor accumulation and high tumor-to-background ratios (Figure 5O–Q). Collectively, these findings underscore the translational potential of this agent for both SPECT/CT diagnostic imaging and targeted radionuclide therapy of PDAC.

#### 4.2.2. Integrin αvβ3

Integrin αvβ3-targeted imaging has evolved from conventional nuclear imaging approaches to advanced multimodal strategies incorporating NIR-II photoacoustic imaging and photothermal therapy. These multifunctional platforms enable simultaneous tumor visualization and treatment, demonstrating the versatility of integrin αvβ3 as a theranostic target in PC.

Jin et al. [175] evaluated the utility of ^99m^Tc-3PRGD_2_ SPECT imaging for monitoring the early response to antiangiogenic therapy in a PANC-1 xenograft model. The results demonstrated a strong correlation between ^99m^Tc and 3PRGD_2_ uptake and microvessel density (MVD). Critically, significant reductions in T/NT were observed as early as day 7 in the Endostar and combination groups, whereas tumor volume measurements showed no significant differences at this early time point. These findings indicate that ^99m^Tc-3PRGD_2_ SPECT can detect therapeutic responses approximately 7–14 days earlier than conventional anatomical assessments. The kit-based formulation of ^99m^Tc-3PRGD_2_ also offers practical advantages for routine clinical use.

Chen et al. [174] developed a cRGD-functionalized cyclo [8]pyrrole nanoparticle (cRGD-CPNP) for NIR-II PA imaging and photothermal therapy (PTT) of pancreatic cancer. In vivo PA tomography revealed distinct tumor distribution patterns between targeted and non-targeted nanoparticles. While control CPNPs accumulated predominantly at the tumor periphery, cRGD-CPNPs penetrated deeply into the tumor core, attributed to cRGD-mediated binding to integrin αvβ3 expressed on both endothelial cells and pancreatic cancer cells. Notably, in an orthotopic pancreatic cancer model, cRGD-CPNP-mediated PTT significantly inhibited intra-abdominal metastases, as confirmed by bioluminescence imaging.

#### 4.2.3. Integrin α6

Integrin α6 has emerged as a promising biomarker characterized by its upregulated expression in pancreatic cancer (PC). Chen et al. [183] developed [^18^F]AlF-NOTA-RD2, an optimized integrin α6-targeted radiotracer synthesized via Good Manufacturing Practice (GMP)-compliant automated procedures. In murine models bearing PANC-1 xenografts, this tracer exhibited significantly superior tumor-to-muscle ratios compared to [^18^F]FDG (8.69 ± 2.03 vs. 1.41 ± 0.23 at 0.5 h post-injection). Furthermore, autoradiographic analysis of human PC tissue specimens corroborated the tracer’s specific accumulation within tumor regions.

#### 4.2.4. Integrin α5

Integrin α5 (ITGA5) is markedly overexpressed within the stromal compartment of PDAC, particularly in activated pancreatic stellate cells (PSCs), which drive the aberrant deposition of ECM proteins. Wang et al. [172] investigated the feasibility of ITGA5-targeted SPECT/CT imaging using ^125^I-AV3, a radiolabeled peptide inhibitor. In vitro studies demonstrated that PSCs conditioned with SW1990 cell supernatants exhibited upregulated ITGA5 expression and enhanced ^125^I-AV3 uptake, as confirmed by Western blotting and immunofluorescence assays. In vivo SPECT/CT imaging of PDAC-bearing nude mice revealed predominant accumulation of ^125^I-AV3 in xenografts established via co-injection of cancer cells and PSCs, whereas tumors derived from cancer cells alone displayed negligible signal intensity. Furthermore, pre-administration of non-radiolabeled AV3 significantly blocked radiotracer uptake in both tumor models, confirming binding specificity. Consistent with these findings, immunohistochemical analysis indicated a substantially higher ITGA5 positivity rate in SW1990/PSC co-implanted tumors compared to those comprising SW1990 cells alone. This study indicates that ^125^I-AV3 is a viable option for SPECT/CT imaging of pancreatic cancer by targeting ITGA5 in pancreatic stellate cells, offering unique stromal insights independent of cancer cell status.

#### 4.2.5. Bispecific Integrin/EGFR Targeting

To address the challenge of tumor heterogeneity, a dual-targeting strategy has been strategically employed. Li et al. [176] engineered [^64^Cu]Cu-NOTA-RGD-GE11, a heterodimeric peptide radiotracer designed to simultaneously target integrin αvβ3 and EGFR. In murine models bearing BxPC3 pancreatic cancer xenografts, this heterodimer demonstrated significantly superior tumor accumulation compared to its monomeric counterparts. Specifically, at 2 h post-injection, tumor uptake reached 4.63 ± 0.25%ID/g for the heterodimer, whereas the monomers [^64^Cu]Cu-NOTA-RGD and [^64^Cu]Cu-NOTA-GE11 exhibited markedly lower values of 1.24 ± 0.18%ID/g and 0.77 ± 0.13%ID/g, respectively. These findings underscore the therapeutic and diagnostic advantages of bispecific targeting in mitigating the effects of intratumoral heterogeneity in pancreatic cancer.

### 4.3. Targeting the Immune Microenvironment

The immunosuppressive tumor microenvironment of PC presents both challenges and opportunities for molecular imaging. Several probes have been developed to visualize immune checkpoint molecules, tumor-infiltrating lymphocytes, and bispecific therapeutic proteins.

#### 4.3.1. PD-L1

PD-L1-targeted imaging strategies have expanded from conventional antibody-based approaches to innovative nanomedicine platforms that enable multimodal visualization and therapeutic intervention.

Xiang et al. [177] evaluated ^68^Ga-HBED-CC-WL12, a small-molecule peptide-based PET radiotracer targeting PD-L1, in a murine PANC02 pancreatic cancer model. Imaging initiated 20 min post-injection enabled effective tumor visualization, with high-contrast images sustained for up to 180 min. Tumor-to-muscle uptake ratios exceeded 2.0 by 90 min, demonstrating the feasibility of rapid, non-invasive imaging of PD-L1 expression in pancreatic cancer.

Zhang et al. [178] developed DCCGP, an organosilica-based nanotheranostic platform integrating fluorescence imaging, MRI, and real-time infrared photothermal imaging for photoimmunotherapy of PC. This system incorporated copper sulfide nanoparticles to enhance tumor penetration and mediate photothermal therapy. The induced hyperthermia triggered immunogenic cell death and alleviated the immunosuppressive tumor microenvironment; when combined with anti-PD-L1 checkpoint blockade, this approach achieved synergistic photoimmunotherapeutic efficacy.

#### 4.3.2. Bispecific T-Cell Engager Targets

Wen et al. [179] constructed ^89^Zr-M17C3, a novel bispecific T-cell engager (BiTE) radiotracer targeting both MUC17 on tumor cells and CD3 on T lymphocytes. Probe uptake was significantly elevated in MUC17-expressing AsPC-1 tumors relative to MUC17-negative PANC-1 controls (SUVmax: 2.26 ± 0.18 vs. 1.13 ± 0.14; *p* < 0.001). In CD3-humanized murine models, the probe enabled the simultaneous visualization of MUC17-positive neoplasms and CD3-expressing immune compartments, thereby establishing a versatile platform for the concurrent assessment of tumor antigen expression and T-cell infiltration.

#### 4.3.3. Cluster of Differentiation

CD-targeted imaging, particularly CD44 and CD47, offers unique insights into tumor–immune interactions in the PC microenvironment. The differential expression patterns of CD markers across tumor subtypes suggest potential applications in patient stratification and personalized treatment approaches.

Liang et al. [180] engineered two VHH-based immunoPET tracers targeting CD47: [^68^Ga]Ga-NOTA-C2, designed for rapid imaging, and [^89^Zr]Zr-DFO-ABDC2, which incorporates an albumin-binding domain to extend systemic circulation half-life. [^68^Ga]Ga-NOTA-C2 effectively delineated pancreatic tumors and discriminated between heterogeneous CD47 expression levels, demonstrating significantly higher uptake in BxPC-3 xenografts (5.40 ± 0.44%ID/g) compared to AsPC-1 xenografts (2.37 ± 0.51%ID/g). In contrast, [^18^F]FDG failed to distinguish between these models, thereby underscoring the superior molecular specificity of CD47-targeted immunoPET imaging.

Tang et al. [190] elucidated a biphasic relationship between serum CA19-9 levels and PDAC malignancy, demonstrating that both CA19-9-negative status and markedly elevated CA19-9 concentrations are associated with increased tumor aggressiveness. Through comprehensive glycoproteomic profiling, the investigators identified CD44 as a significantly upregulated biomarker specific to CA19-9-negative PDAC, thereby enabling its differentiation from both CA19-9-positive tumors and benign pancreatic conditions. Leveraging these insights, the team developed an ^89^Zr-labeled anti-CD44 monoclonal antibody (^89^Zr-1M2E3) for PET/CT imaging, which exhibited robust targeting efficacy against CA19-9-negative tumors in preclinical models. Collectively, these findings establish CD44 as a promising diagnostic biomarker and therapeutic target for the clinically challenging CA19-9-negative PDAC subtype.

#### 4.3.4. Bispecific EGFR/VEGF Theranostic Protein

Wang et al. [182] developed Bi-fp50, a compact bispecific protein targeting both EGFR and VEGF to enhance tumor penetration and treat PDAC. They engineered Bi-fp50 using synthetic biology and labeled it with fluorescent dyes for in vitro and in vivo imaging. At low concentrations, Bi-fp50 strongly inhibited proliferation of BxPC3 and AsPC1 PDAC cells, showing synergistic therapeutic effects superior to the single scFv controls in vitro. Its blood clearance half-life was 4.33 ± 0.23 h. After intravenous injection, Bi-fp50 progressively infiltrated tumors, achieving widespread intratumoral distribution and accumulating nearly twice as much as scFv2 in an orthotopic PDAC model, as shown by near-infrared fluorescence imaging and confocal microscopy. Bi-fp50 induced widespread tumor apoptosis and suppressed tumor growth for three weeks in vivo without notable side effects. This study suggests Bi-fp50 as a promising tumor suppressor and an effective, safe theranostic agent for PDAC.

### 4.4. Other Molecular Targets

#### 4.4.1. Trop2

Trop2-targeted imaging has emerged as a promising theranostic strategy for pancreatic cancer, with both antibody-based immunoPET and radioligand therapy demonstrating significant preclinical efficacy. The continued development of humanized antibodies and optimized radiolabeling methods is expected to accelerate clinical translation.

Chen et al. [183] developed ^89^Zr-DFO-AF650, an anti-Trop2 antibody immunoPET probe. In BxPC-3 xenografts, tumor uptake reached 28.8 ± 7.63%ID/g at 120 h, compared to 6.76 ± 2.08%ID/g in MIA PaCa-2 and 3.51 ± 0.69%ID/g in AsPC-1 (*p* < 0.0001). In an orthotopic model, the probe clearly visualized primary tumors with uptake of 26.0 ± 6.43%ID/g versus normal pancreatic uptake of only 3.20 ± 1.40%ID/g, and detected tumors as small as approximately 45 mm^3^.

Li et al. [184] further developed a theranostic pair, ^64^Cu-NOTA-hIMB1636 for PET imaging and ^177^Lu-DOTA-hIMB1636 for radioligand therapy. PET imaging demonstrated specific Trop2-dependent tumor visualization (48 h uptake: 8.95 ± 1.07%ID/g), and ^177^Lu-labeled antibody therapy significantly suppressed Trop2-positive tumor growth, demonstrating the theranostic potential of this approach.

#### 4.4.2. EGFR

Epidermal Growth Factor Receptor (EGFR) is overexpressed in up to 90% of PC cases, establishing it as a paramount target for molecular imaging strategies. Recently, a novel immuno-PET approach using ^64^Cu-NCAB001 administered intraperitoneally (ipPET) was developed to detect orthotopic pancreatic tumor xenografts as small as 3 mm in murine models [185]. Safety and biodistribution assessments in cynomolgus monkeys demonstrated that ultrasound-guided intraperitoneal administration of ^64^Cu-NCAB001 was well-tolerated, with no observed adverse events. PET imaging revealed rapid clearance of radioactivity from the peritoneal cavity within 6 h, resulting in negligible background activity surrounding the pancreas by 24 h, thereby yielding high-contrast images. Furthermore, the estimated human effective dose was comparable to that of standard ^18^F-FDG PET.

A novel formulation comprising an acetate buffer, glycine, and polysorbate 80 was developed to facilitate clinical translation. This excipient system enables the long-term storage of the antibody–chelator conjugate for up to one year and ensures the radiochemical stability of ^64^Cu-NCAB001 for at least 24 h post-radiolabeling, thereby maintaining radioactivity concentrations sufficient for clinical administration [186]. Collectively, these findings substantiate the advancement of ^64^Cu-NCAB001 immunoPET into clinical trials as a promising EGFR-targeted imaging modality for the early detection of PC.

#### 4.4.3. MUC

The MUC gene encodes transmembrane or secreted mucins, which are involved in epithelial protection, signal transduction, and tumor development. Mucin-targeted imaging strategies for MUC5AC, MUC16, and MUC4 demonstrate the potential of exploiting differential mucin expression patterns for PC diagnosis.

MUC5AC is aberrantly expressed in pancreatic cancer compared to the normal pancreas and chronic pancreatitis. Henry et al. [187] developed ^89^Zr-DFO-RA96, an anti-MUC5AC antibody immunoPET probe. In Capan-2 xenografts, tumor uptake reached 14.6 ± 1.5%ID/g at 144 h, significantly higher than blocking controls (6.6 ± 1.5%ID/g) and IgG controls (8.8 ± 0.3%ID/g). In situ pancreatic cancer models with varying MUC5AC expression showed probe uptake proportional to MUC5AC levels, confirming target specificity [210]. Nakata et al. [188] developed a humanized anti-MUC5AC antibody-based theranostic pair, NMK89 (^89^Zr-labeled for diagnosis) and NMT25 (^225^Ac-labeled for alpha-therapy). NMK89 demonstrated high tumor accumulation (22.0 ± 3.7%ID/g at 68 h), while NMT25 showed dose-dependent antitumor efficacy, establishing a promising theranostic platform for MUC5AC-positive PC.

MUC4 is a mucin significantly expressed in PDAC, with minimal presence in the normal pancreas. Jaiswal et al. [198] developed anti-MUC4-IRDye800CW, a near-infrared fluorescently labeled MUC4 antibody for intraoperative imaging. In orthotopic mouse models, the probe effectively highlighted liver metastases and peritoneal carcinomatosis, successfully labeling metastatic lesions as small as 1 mm. These findings support the potential of MUC4-targeted NIRF imaging for intraoperative staging and margin assessment in pancreatic cancer surgery.

#### 4.4.4. ACE2

Angiotensin-converting enzyme 2 (ACE2), a crucial regulator of the renin–angiotensin–aldosterone system (RAAS), is notably upregulated in PC and acts as a potential anticancer agent by inhibiting angiogenesis and invasion. Zhou et al. [190] created [^99m^Tc]Tc-cyc-DX600, an SPECT imaging tracer specifically targeting ACE2 for PC. The probe demonstrated specific accumulation in ACE2-positive xenografts, with blocking experiments confirming target specificity. In orthotopic KPC models, ACE2 SPECT uptake positively correlated with tumor volume and ACE2 immunohistochemical expression, while showing a negative correlation with ^18^F-FDG PET uptake. These findings establish [^99m^Tc]Tc-cyc-DX600 SPECT as a promising functional imaging modality for non-invasively evaluating ACE2 expression status in PC, with potential clinical translational value for patient stratification and treatment planning.

#### 4.4.5. EphA2

EphA2, a receptor tyrosine kinase, is overexpressed in PDAC and associated with aggressive tumor biology. Sharma et al. [191] developed a theranostic platform comprising [^68^Ga]Ga-AJ201 for PET imaging and [^225^Ac]Ac-AJ210 for targeted alpha-particle therapy. [^68^Ga]Ga-AJ201 achieved high-contrast imaging in multiple PDAC models with uptake reflecting EphA2 expression levels. [^225^Ac]Ac-AJ210 demonstrated dose-dependent cytotoxicity and significantly inhibited tumor growth while prolonging survival in KPC syngeneic models, establishing EphA2 as a promising radiotheranostic target.

#### 4.4.6. c-Met

Hepatocyte growth factor receptor (c-Met) is overexpressed in approximately 84% of PDAC cases. To leverage this molecular target, Sun et al. [192] developed [^68^Ga]Ga-NOTA-PFCM01, a nanobody-based radiotracer designed for c-Met-specific immunoPET imaging. In BXPC-3 xenograft models, the tracer demonstrated a tumor uptake of 2.31 ± 0.02%ID/g at 2 h post-injection. Notably, PET imaging studies in healthy cynomolgus monkeys revealed rapid renal clearance and minimal background accumulation in critical organs, including the liver, gastrointestinal tract, pancreas, and skeletal muscle. These findings underscore the tracer’s favorable pharmacokinetic profile and support its potential for clinical translation.

#### 4.4.7. CSPG4

Chondroitin sulfate proteoglycan 4 (CSPG4) is highly expressed on various solid tumor cells and cancer stem cells. Wang et al. [193] evaluated three ^68^Ga-labeled CSPG4-targeting peptide PET probes. Among them, [^68^Ga]Ga-DOTA-TH10 demonstrated the most favorable in vivo characteristics, with ASPC-1 pancreatic tumor uptake of 1.63 ± 0.15%ID/g at 60 min that was significantly reduced by blocking (0.88 ± 0.09%ID/g) and low uptake in CSPG4-negative HepG2 tumors (0.60 ± 0.05%ID/g), confirming CSPG4-mediated specificity.

#### 4.4.8. Hsp90

Heat shock protein 90 (Hsp90) is a molecular chaperone overexpressed in PDAC compared to normal or inflamed tissues, making it a promising target for discriminating malignancy from benign inflammatory masses. Wang et al. [194] developed ^18^F-NOTA-Dimer-Sansalvamide A (^18^F-NOTA-Dimer-San A), an Hsp90-targeted PET probe. The probe demonstrated high and specific uptake in PL45 xenograft tumors (5.80 ± 0.94%ID/g at 2 h), with significantly lower accumulation in turpentine-induced inflammatory muscle (1.47 ± 0.42%ID/g, *p* < 0.05), yielding a favorable tumor-to-inflammation ratio of 3.60 ± 1.80, supporting its potential as a PET tracer for improving specificity beyond ^18^F-FDG in PC diagnosis.

#### 4.4.9. MT1-MMP

Membrane-type 1 matrix metalloproteinase (MT1-MMP) is involved in extracellular matrix degradation and tumor invasion. Yang et al. [195] developed [^68^Ga]Ga-DOTA-BT1718 for MT1-MMP PET imaging. The probe demonstrated high uptake in MT1-MMP-expressing Capan-2 pancreatic tumors (5.11 ± 0.21%ID/g at 30 min) and predominant urinary clearance with low background. Preliminary clinical translation in pancreatic cancer patients showed significant tumor uptake with SUVmax approximately 6.6, supporting its potential for clinical application.

#### 4.4.10. ST14

ST14 (matriptase) is a type II transmembrane serine protease that is characteristically overexpressed in PC. Addressing the limitation of elevated false-positive rates associated with ^18^F-FDG PET in the differential diagnosis of pancreatitis, Peng et al. [196] developed a novel ^68^Ga-labeled radiopharmaceutical specifically targeting ST14. Subsequent radiolabeling yielded ^68^Ga-ST14-06 with high radiochemical purity and robust stability. Comprehensive in vitro and in vivo evaluations, including cellular uptake assays and PET/CT imaging in AsPC-1 tumor-bearing murine models, confirmed specific ST14 engagement. Notably, the tracer demonstrated significantly enhanced accumulation in ST14-positive tumors, while signal intensity was markedly reduced upon competitive blocking. These preliminary findings position ^68^Ga-ST14-06 as a pioneering PET tracer for the noninvasive molecular imaging of pancreatic cancer via targeted ST14 visualization.

#### 4.4.11. 5T4

5T4 is a 72 kDa transmembrane glycoprotein with low normal tissue expression but significant upregulation in PC. Kong et al. [197] developed [^18^F]AlF-RESCA-H006, a single-domain antibody fragment-based PET probe targeting 5T4. The tracer clearly visualized tumors in both BxPC-3 (2.43 ± 0.46%ID/g) and PANC-1 (2.88 ± 1.02%ID/g) xenograft models at 60 min, establishing proof-of-concept for 5T4-targeted PET imaging in PC.

#### 4.4.12. PARP-1

Poly ADP-ribose polymerase-1 (PARP-1) is a key enzyme in DNA damage repair and an important therapeutic target. Gao et al. [198] developed PEG-modified DOTA-PEG-PARPi labeled with ^68^Ga for PET imaging and ^177^Lu for radioligand therapy. The physicochemical properties of the PARPi probes were optimized through polyethylene glycol modification, demonstrating sufficient in vivo stability. In PSN-1 pancreatic cancer models, ^177^Lu-DOTA-PEG-PARPi demonstrated significant tumor growth inhibition. The PEG modification improved upon previous ^18^F-labeled PARP probes by reducing hepatobiliary background and enabling better detection of abdominal lesions.

Patel et al. [199] synthesized [^18^F]AZD9574, an advanced PET radiotracer exhibiting high selectivity for poly(ADP-ribose) PARP-1 and capable of crossing the blood–brain barrier. Evaluations across a diverse panel of breast, glioblastoma, prostate, and pancreatic cancer cell lines revealed that tracer uptake correlated strongly with endogenous PARP-1 expression levels. Furthermore, this uptake was inhibited in a dose-dependent manner by several clinically relevant PARP inhibitors, thereby confirming its binding specificity. In 22Rv1 xenograft mouse models, [^18^F]AZD9574 exhibited significant tumor accumulation attributable to specific PARP-1 targeting. Ex vivo biodistribution studies corroborated these findings, demonstrating organ uptake profiles consistent with specific tumor binding and PARP-1 expression patterns. Collectively, [^18^F]AZD9574 emerges as a promising PARP-1-targeted imaging agent with substantial potential to advance oncological research and therapeutic monitoring. It facilitates the precise quantification of PARP-1 expression, real-time assessment of target engagement, and the evaluation of treatment response.

#### 4.4.13. uPAR

Urokinase plasminogen activator receptor (uPAR) is overexpressed in PC and associated with tumor invasion and metastasis. The application of uPAR immunoPET for assessing treatment-induced senescence represents an innovative approach to monitoring therapeutic response beyond conventional anatomical imaging.

Luo et al. [200] developed uPAR-ICG-FVIOs, a multimodal nanoplatform comprising uPAR-targeted, indocyanine green (ICG)-conjugated ferrimagnetic vortex iron oxide nanorings for dual NIRF/MR imaging combined with magnetic hyperthermia therapy [194]. The system demonstrated effective tumor targeting and enabled imaging-guided therapeutic intervention.

Pratt et al. [201] developed ^89^Zr-labeled anti-uPAR antibody immunoPET for assessing treatment-induced senescence. In MiaPaCa-2 xenografts treated with trametinib and palbociclib (TP), ^89^Zr-DFO-anti-huPAR tumor uptake significantly increased after therapy, suggesting uPAR as a potential imaging biomarker for therapy-induced senescence in PC. Immuno-PET imaging using anti-uPAR antibodies demonstrated greater tumor selectivity compared to [^18^F]F-FDG or [^18^F]F-DPA-714, positioning radiolabeled uPAR-targeting antibodies as promising candidates for PET imaging and monitoring chemotherapy-induced senescence.

#### 4.4.14. Cell Surface Glycans

Tumor-specific glycosylation represents a distinct class of imaging targets. Kuroda et al. [202] developed [^18^F]FB-rBC2LCN, targeting the H-type-3 fucosylated glycan structure aberrantly expressed on PDAC cell surfaces. In Capan-1 tumor-bearing mice, tumor uptake progressively increased from 6.6 ± 1.8%ID/g at 60 min to 11.0 ± 3.2%ID/g at 240 min, with tumor-to-muscle ratios reaching 19.0 ± 1.8 at 360 min, demonstrating excellent tumor contrast accumulation over time.

#### 4.4.15. Glutamine/KRAS

Given that over 90% of PDACs harbor KRAS mutations and different mutation subtypes exhibit varying glutamine dependency, glutamine metabolism imaging has emerged as a strategy for non-invasive molecular subtyping. Liu et al. [203] utilized (2S,4R)-4-[^18^F]FGln PET to distinguish KRAS G12D (PANC-1) from G12C (MIA PaCa-2) mutant tumors. PANC-1 tumors demonstrated significantly higher probe uptake at all time points (30 min SUVmax: 1.03 ± 0.02 vs. 0.41 ± 0.02; 60 min: 0.92 ± 0.05 vs. 0.43 ± 0.03), whereas [^18^F]FDG showed no significant difference between the models, suggesting that (2S,4R)-4-[^18^F]FGln PET may enable non-invasive detection of KRAS mutation subtypes to guide targeted therapy.

#### 4.4.16. NTSR1

Neurotensin receptor subtype 1 (NTSR1) is overexpressed in at least 75% of PDAC. Potemkin et al. [204] developed novel ^99m^Tc-labeled non-peptide NTSR1 antagonists for SPECT imaging. The lead compound [^99m^Tc]Tc-HYNIC complex demonstrated excellent radiochemical yield (94–96%), stability in human serum and plasma, and high specific tumor uptake (12 ± 2.8%ID/g at 4 h) with favorable tumor-to-background ratios in HT-29 xenografts, establishing a promising SPECT imaging agent for NTSR1-positive tumors.

#### 4.4.17. GRPR

The Gastrin-Releasing Peptide Receptor (GRPR) is markedly overexpressed in PC, establishing it as a promising target for multimodal nuclear and optical imaging. Tu et al. [205] developed GB-6, a novel GRPR-targeting peptide dual-labeled with ^99m^Tc for SPECT and an NIR fluorophore, MPA, for intraoperative optical guidance. The radiotracer [^99m^Tc]Tc-HYNIC-PEG4-GB-6 demonstrated favorable biodistribution profiles one-hour post-injection, characterized by high tumor-to-muscle and tumor-to-bone uptake ratios alongside predominant renal clearance. Concurrently, the NIR probe MPA-PEG4-GB-6 yielded robust tumor-to-pancreas fluorescence signal ratios of 5.2 ± 0.3 in subcutaneous xenograft models and 7.66 ± 0.48 in orthotopic models, thereby enabling precise tumor delineation to facilitate image-guided surgical navigation.

#### 4.4.18. NPC1L1

Niemann–Pick C1-like 1 (NPC1L1), a cholesterol transporter, is recognized as a target for imaging and modulating the microenvironment in PC. Dong et al. [206] introduced CHOL@GEM-NBs, nanobubbles with a cholesterol-based shell designed to deliver gemcitabine by targeting NPC1L1. The system demonstrated superior ultrasound molecular imaging capabilities, characterized by increased contrast intensity and prolonged duration. Utilizing ultrasound-targeted nanobubble destruction (UTND), this method facilitated targeted drug delivery, mitigated NPC1L1-mediated cholesterol hijacking, induced immunogenic cell death, and restructured the immunosuppressive tumor microenvironment, offering a novel integrated strategy for pancreatic cancer diagnosis and chemoimmunotherapy.

#### 4.4.19. CA19-9

CA19-9 serves as the predominant serum biomarker for the diagnosis of PC. Chen et al. [207] developed TE-1132, a CA19-9-targeted single-chain variable fragment-Fc (scFv-Fc) antibody construct, site-specifically conjugated with six DOTA chelators to facilitate theranostic applications. The scFv-Fc architecture confers enhanced tumor penetrability within solid malignancies owing to its reduced molecular dimensions, while maintaining favorable pharmacokinetic properties. Furthermore, a chelator-to-antibody ratio of six enables the radiolabeling of TE-1132 with lutetium-177 (^177^Lu) to achieve high specific activities. In murine models bearing BxPC3 xenografts, immuno-SPECT/CT imaging utilizing [^111^In]In-TE-1132 demonstrated selective tumor accumulation. Therapeutic administration of [^177^Lu]Lu-TE-1132, delivered via single or fractionated dosing regimens, significantly suppressed BxPC3 tumor progression and prolonged survival without inducing irreversible body weight loss or hematopoietic toxicity. Collectively, the favorable pharmacokinetic profile, robust tumor uptake, and efficacy coupled with an acceptable safety margin support the further clinical translation of the theranostic agent TE-1132 for the management of pancreatic cancer.

Cox et al. [208] engineered a CA19-9-targeted NIRF probe, CA19-9-IRDye800CW, to facilitate fluorescence-guided surgery for PC. In orthotopic murine models utilizing SW1990 and BxPC3 cell lines, the immunoconjugate exhibited significantly superior tumor-to-pancreas and tumor-to-liver contrast ratios compared to non-specific IgG-IRDye800CW controls. Notably, effective tumor visualization was achieved using the SPY fluorescence laparoscope, an FDA-cleared clinical imaging system. Furthermore, the absence of significant probe accumulation in CA19-9-negative MIA PaCa-2 tumors corroborated the agent’s target specificity. Collectively, these findings underscore the translational potential of antibody-based NIRF imaging for the intraoperative detection of pancreatic malignancies when integrated with established clinical imaging platforms.

#### 4.4.20. CEA

Van Manen et al. [209] developed NbCEA5-ZW800-1, a carcinoembryonic antigen-targeting nanobody conjugated to a zwitterionic NIR dye for fluorescence-guided surgery. In orthotopic PC models, the probe accumulated specifically in tumors with a mean in vivo tumor-to-background ratio of 2.4, demonstrating the feasibility of nanobody-based same-day injection protocols for intraoperative imaging.

#### 4.4.21. TLR2

Toll-like receptor 2 (TLR2) is broadly expressed among PC. Huynh et al. [210] developed TLR2L-800, a high-affinity fluorescent molecular imaging probe targeting TLR2, designed to facilitate intraoperative tumor detection and margin assessment. In an orthotopic human pancreatic xenograft model, three experimental cohorts were administered TLR2L-800: one underwent fluorescence-guided surgery (FGS), another received visible light surgery (VLS), and the third served as a non-surgical control. At 24 h post-administration, the FGS cohort underwent real-time fluorescence image-guided tumor resection. Survival analysis at 41 days post-operatively revealed a 53% survival rate in the FGS group, whereas both the VLS and non-surgical groups exhibited 0% survival. Furthermore, the FGS group demonstrated a significantly improved long-term survival rate of 35% at 200 days, compared to 0% in the VLS group (*p* = 0.0018). This study highlights the potential to enhance disease-free survival in PC patients by achieving R0 margins through TLR2-targeted fluorescence molecular imaging, introducing a novel lipopeptide ligand-based method for FGS.

#### 4.4.22. MCT

Monocarboxylate transporters (MCTs), particularly MCT1 and MCT4, are overexpressed in various malignancies, including PC. Shi et al. [211] developed [^18^F]FEtO-CHC, a novel α-cyano-4-hydroxycinnamic acid-derived PET radiotracer targeting MCTs. In vitro studies in MCT1-expressing BxPC3 pancreatic cancer cells demonstrated MCT-dependent uptake with >70% suppression by the MCT inhibitor α-CHC. Dynamic micro-PET/CT imaging in tumor-bearing mice revealed detectable tumor uptake that correlated with MCT1 expression levels, with dual hepatorenal clearance as the primary excretion pathway. These findings validate [^18^F]FEtO-CHC as an MCT-targeted PET probe and support its potential utility in non-invasive imaging of MCT-positive tumors, though further structural optimization is warranted to enhance tumor uptake and reduce background retention.

#### 4.4.23. Folate Receptor

The folate receptor (FR) is overexpressed in PC, where it supports rapid cell proliferation by facilitating folate uptake for nucleotide biosynthesis. Lee et al. [212] developed an innovative liposomal PET imaging strategy that combines FR-targeted delivery with esterase-responsive background clearance to achieve ultrahigh tumor-to-background contrast. A folate-conjugated liposomal nanoplatform encapsulating the esterase-labile radiotracer [^124^I]HIB was engineered to specifically target FR-overexpressing pancreatic tumors. In orthotopic PC models, PET imaging facilitated the distinct visualization of millimeter-scale lesions as early as 4 h post-injection. By 24 h, tumor accumulation exceeded 9% of the injected dose, yielding superior tumor-to-background ratios that enabled high-contrast imaging with negligible off-target interference. This dual-targeting strategy, which leverages both receptor-mediated endocytosis and microenvironment-responsive activation, effectively mitigates the persistent challenge of non-specific sequestration by the mononuclear phagocyte system (MPS). Consequently, this approach represents a promising paradigm for the early detection of PC.

### 4.5. Stimuli-Responsive Polymer

The strategic integration of stimuli-responsive mechanisms and active targeting moieties into nanoparticle platforms has heralded a paradigm shift in the precision imaging and therapeutic delivery for PC. This synergistic approach facilitates spatiotemporally controlled contrast enhancement and payload release, effectively navigating the complexities of the tumor microenvironment (TME).

#### 4.5.1. Enzyme-Responsive Polymer

Enzyme-activatable molecular imaging represents an innovative strategy for cancer detection that leverages the dysregulated enzymatic activity within the tumor microenvironment to achieve high signal-to-background contrast. These smart agents remain quiescent until encountering tumor-specific enzymes that trigger their activation through cleavage, reduction, or conformational changes. PC exhibits distinct enzymatic alterations, including upregulated peptidases, reductases, and lysosomal hydrolases, which provide a biochemical basis for selective probe activation.

Takahashi et al. [213] developed an innovative enzyme-activatable fluorescence imaging strategy using the GP-HMRG probe. Through screening of 309 unique fluorescence probes against tumor and non-tumor lysates, GP-HMRG was selected as optimal for PC detection. Spray application onto resected specimens enabled rapid fluorescence visualization of cancer tissue with high tumor-to-background ratios. Mechanistically, dipeptidyl peptidase IV (DPP-IV) or a DPP-IV-like enzyme was identified as the tumor-specific activator, providing a rapid, real-time method for intraoperative tissue diagnosis.

In an innovative approach combining nanomedicine with targeted radionuclide therapy, Su et al. [214] developed ^125^I-TiO_2_-TAT/HA2, a lysosome-escaping and nucleus-targeting nanomedicine for enhanced cancer catalytic internal radiotherapy (CIR) of PC. Confocal microscopy and biological transmission electron microscopy confirmed the nucleus-targeting ability of TiO_2_-TAT/HA2. In vitro, ^125^I-TiO_2_-TAT/HA2 significantly increased apoptosis and DNA damage and reduced DNA repair protein expression (RAD51 and 53BP1) versus ^125^I-TiO_2_. In vivo, SPECT imaging confirmed prolonged intra-tumoral retention of ^125^I-TiO_2_-TAT/HA2. Tumors treated with ^125^I-TiO_2_-TAT/HA2 displayed significantly smaller volume, enhanced necrosis and apoptosis, and downregulated DNA repair protein expression compared with ^125^I-TiO_2_. This strategy presents a promising method to improve tumor therapy through precise nuclear delivery of reactive oxygen species rather than increased ROS production, and could be adapted for other macromolecular therapeutics.

In a pioneering advancement integrating nanomedicine with targeted radionuclide therapy, Su et al. [215] developed ^125^I-TiO_2_-TAT/HA2, a multifunctional nanoplatform engineered to facilitate lysosomal escape and achieve precise nuclear localization. This design aims to augment the efficacy of catalytic internal radiotherapy (CIR) for PC. In vitro studies demonstrated that, relative to the control formulation (^125^I-TiO_2_), ^125^I-TiO_2_-TAT/HA2 significantly potentiated apoptosis and induced severe DNA damage, concomitant with a marked downregulation of key DNA repair proteins, specifically RAD51 and 53BP1. In vivo, SPECT imaging corroborated the prolonged intratumoral retention of the nanocomplex. Therapeutically, tumors treated with ^125^I-TiO_2_-TAT/HA2 exhibited substantial volume regression, extensive necrosis, and elevated apoptotic indices, alongside suppressed expression of DNA repair markers compared to those receiving ^125^I-TiO_2_. This strategy offers a promising approach to enhance tumor therapy by precisely delivering reactive oxygen species to the nucleus, rather than merely increasing ROS production, and it may be applicable to other macromolecular therapeutics.

#### 4.5.2. pH/GSH-Responsive Polymer

**Figure 6:**
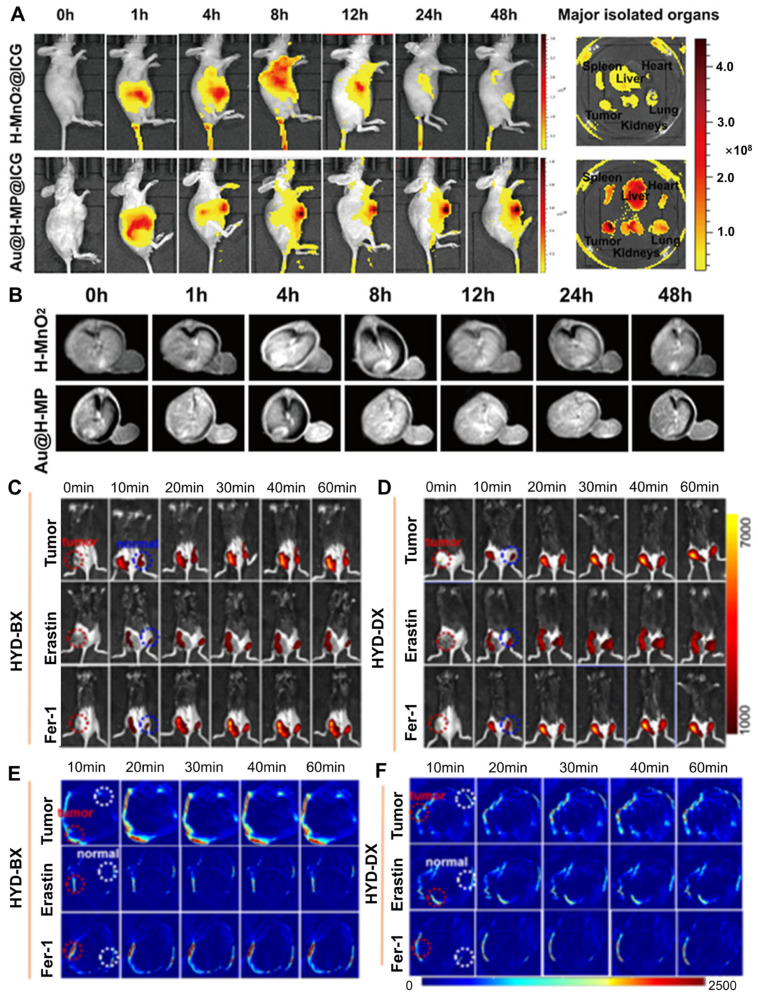
Dual-responsive polymer for pancreatic cancer imaging. Fluorescence images (**A**) and MR images (**B**) of nude mice at 0–48 h. Reproduced from ref. [17], Copyright 2025, American Chemical Society. Real-time fluorescence (**C**,**D**) and photoacoustic (**E**,**F**) imaging of endogenous Cys/GSH during ferroptosis in Panc02 tumor-bearing mice using HYD-BX or HYD-DX. Reproduced from ref. [15], Copyright 2024, American Chemical Society.

Zhang et al. [17] engineered a dual-responsive, Janus-structured Au@H-MP@DOX nanomotor capable of molecular MRI, specifically designed for the synergistic photothermal-chemotherapeutic treatment of PC. These Au@H-MP nanomotors (NMs) were fabricated via magnetron sputtering, with DOX selectively loaded onto one hemisphere to facilitate combined therapeutic modalities. The system exhibits sensitivity to both acidic pH and elevated GSH levels, thereby enabling precise, stimuli-triggered drug release within the deep tumor microenvironment. Comprehensive evaluations confirmed the nanomotor’s biocompatibility, high photothermal conversion efficiency, and potent capacity to induce apoptotic cell death. In vitro migration and infiltration assays validated its superior penetrability into tumor tissues, while in vivo studies demonstrated significant tumor suppression accompanied by enhanced intratumoral drug accumulation. Furthermore, the integration of MR and fluorescence imaging modalities allowed for real-time monitoring of nanomotor dynamics, establishing a promising paradigm for image-guided, multimodal therapy against PC (Figure 6A,B).

#### 4.5.3. Cys/GSH-Responsive Polymer

In PDAC, depletion of biothiols, particularly cysteine (Cys) and GSH, is a well-established upstream metabolic hallmark of ferroptosis, directly linking redox imbalance to therapy resistance and tumor vulnerability. To exploit this target for molecular imaging, Li et al. [15] developed two biothiol-activatable NIR fluorescent/photoacoustic bimodal probes, HYD-BX (for Cys) and HYD-DX (for GSH), for real-time visualization of ferroptosis in PDAC (Figure 6C,D). In erastin-induced ferroptotic Panc02 cells, both probes showed significantly attenuated signals due to biothiol depletion, whereas pretreatment with the ferroptosis inhibitor Fer-1 preserved the fluorescence and PA signals. In Panc02 tumor-bearing mice, intratumoral injection of the probes enabled clear bimodal imaging of endogenous Cys and GSH levels, with signal reduction effectively reporting ferroptotic status (Figure 6E,F). Furthermore, the probes successfully imaged cisplatin-induced ferroptosis in vivo, validating their utility as functional imaging tools for monitoring biothiol dynamics during ferroptosis and advancing the understanding of Cys depletion-induced pancreatic tumor ferroptosis.

#### 4.5.4. Other Stimuli-Responsive Polymer

Amaolo et al. [216] engineered hybrid PLGA-phospholipid nanoparticles incorporating gadolinium chelates, functionalized with albumin, adenosine, or glutamine, to improve MRI of PDAC. Hybrid PLGA-lipid nanoparticles were created via an oil-in-water emulsion solvent extraction technique, utilizing DSPE-PEG(2000) and DPPE-PEG(2000)-NHS for surface functionalization. In vitro experiments with MiaPaca2 and Panc1 cell lines demonstrated that functionalized nanoparticles exhibited superior cellular uptake and enhanced MRI signal compared to unconjugated controls, with albumin-PLGA-lipid nanoparticles achieving the highest uptake. All preparations exhibited stable characteristics, a hydrodynamic diameter under 200 nm, and a mildly negative surface charge, indicating strong potential for cellular and in vivo applications. These findings suggest a promising approach for enhancing the sensitivity and specificity of MRI in both diagnosing and treating PDAC.

## 5. Integrative Summary of Imaging Strategies

**Figure 7:**
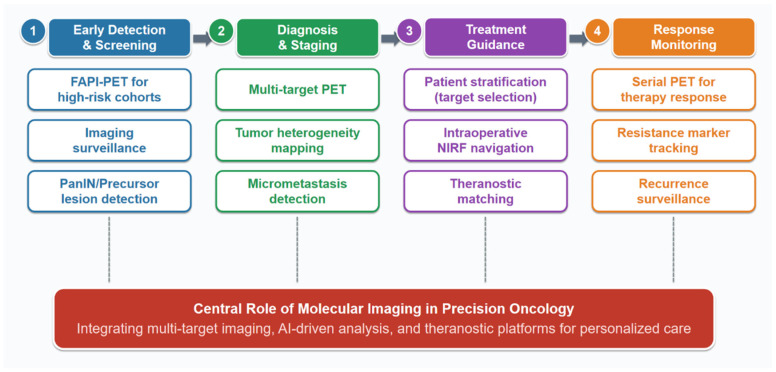
Molecular imaging-guided precision management of pancreatic cancer.

Molecular imaging-guided precision management of pancreatic cancer encompasses a continuum of clinical applications spanning early detection, accurate diagnosis, treatment guidance, and response monitoring (Figure 7). In the early detection setting, FAPI-PET and CA199-targeted imaging modalities are being evaluated for surveillance of high-risk cohorts and identification of precursor lesions. For diagnosis and staging, multi-target PET imaging combining FAP and integrin tracers enables comprehensive tumor characterization and micrometastasis detection. During treatment, patient stratification based on target expression profiling, intraoperative NIRF navigation, and theranostic matching of radioligand therapy to imaging-positive lesions are transforming therapeutic decision-making. Post-treatment, serial molecular imaging facilitates early assessment of therapy response, tracking of resistance marker dynamics, and surveillance for disease recurrence, thereby supporting adaptive treatment strategies throughout the disease trajectory.

To realize this continuum, it is essential to understand the diversity of current molecular imaging probes, which differ substantially in biological specificity, imaging performance, and translational maturity. Probes targeting cell-surface proteins such as EGFR, Trop2, c-Met, and 5T4 provide improved molecular selectivity and enable patient stratification, although their performance can be affected by intratumoral heterogeneity and variable expression levels. Stromal and microenvironment-directed agents, exemplified by FAP-targeted tracers, demonstrate high tumor-to-background contrast and promising theranostic potential with radioligand therapy applications, yet remain limited by uptake in inflammatory and fibrotic conditions. Emerging probes against immune checkpoint markers (e.g., PD-L1, CD47), tumor-associated glycans, and metabolic pathways (e.g., glutamine/KRAS) offer opportunities for therapy monitoring and early detection but are still largely in preclinical development.

Designing probes necessitates balancing tumor retention with imaging kinetics. Full-length antibodies, like trastuzumab and cetuximab derivatives, offer high binding capacity and prolonged tumor retention. However, they are hindered by slow blood clearance, significant hepatic background, and extended circulation times, requiring delayed imaging (48–96 h). Low-molecular-weight agents such as affibodies, nanobodies, peptides, and small molecules provide quick tissue penetration and high contrast within hours. However, optimizing their clinical use for abdominal imaging is necessary to reduce high renal retention, which can obscure local metastases. Innovative formats such as bispecific T-cell engager radiotracers and scFv-Fc fusion proteins represent emerging solutions that balance tumor penetration with favorable pharmacokinetics.

Regarding theranostic translation, a clear distinction exists between approaches ready for clinical implementation and those requiring further exploration. Radionuclide therapies targeting established biomarkers, such as FAP (^177^Lu-FAPI-46) and integrin αvβ6 (^177^Lu-DOTA-5G), offer a direct route to clinical application by utilizing current regulatory frameworks. In contrast, complex nanoplatforms integrating magnetic hyperthermia, photothermal therapy, or stimuli-responsive drug release remain largely exploratory. Although promising for synergistic multimodal treatment, these nanoprobes encounter major challenges in large-scale production, consistency, and long-term toxicity evaluation, making them more suitable as future solutions rather than immediate clinical options.

## 6. Challenges and Future Perspectives

### 6.1. Current Challenges

The significant advancements in targeted molecular imaging for pancreatic cancer highlighted earlier emphasize the transformative potential of this field. Although the value of molecular imaging in pancreatic cancer is increasingly acknowledged, its broad clinical adoption is hindered by several significant challenges that need to be overcome to fully harness its potential.

The spatiotemporal variability in target expression continues to pose a significant challenge. Biomarkers like FAP, integrins, Trop2, and PD-L1 show dynamic changes in both primary and metastatic tumors, affected by the tumor microenvironment, treatment, and disease progression [217]. Imaging focused on a single target can lead to false negatives or underestimated expression levels, thereby reducing diagnostic accuracy and affecting therapeutic decisions. Developing multiplexed imaging strategies that simultaneously evaluate multiple complementary targets may help mitigate this limitation.

Second, probe performance remains affected by unresolved pharmacokinetic limitations and nonspecific uptake. Full-length antibodies have extended circulation times and delayed optimal imaging windows, whereas nanobodies and small peptides tend to accumulate in the kidneys, potentially obscuring abdominal metastases. Certain small-molecule tracers also exhibit elevated background uptake in inflammatory tissues. Structural optimization strategies, including incorporation of albumin-binding domains, hydrophilic linker designs, and construction of dual-target heterodimers, may improve pharmacokinetics and enhance tumor specificity.

Third, the biological safety of novel imaging agents, particularly nanoparticle-based platforms, represents a critical concern requiring systematic evaluation. Nanomaterials may trigger immunological responses, complement activation, or off-target organ accumulation, necessitating comprehensive preclinical toxicology studies before human administration. Long-term biosafety data for stimuli-responsive and theranostic nanoplatforms remain largely unavailable, representing a significant gap that must be addressed through rigorous standardized testing protocols.

Fourth, each imaging modality carries inherent limitations that constrain clinical applicability. PET imaging requires short-lived radioisotopes produced in costly cyclotron facilities, while optical imaging is restricted by tissue penetration depth. Quantitative accuracy varies significantly across platforms, and the lack of standardized acquisition parameters, uptake times, and reporting criteria limits reproducibility and hinders multicenter trial conduct [218]. Establishing consistent imaging protocols and quantitative benchmarks is essential for translating molecular imaging into routine clinical practice.

Finally, the integration of artificial intelligence with molecular imaging faces critical challenges, including limited annotated datasets, annotation bias, and lack of model generalizability across diverse patient populations [219]. Regulatory frameworks for AI-assisted molecular imaging remain underdeveloped, and validation requirements for clinical deployment are still being defined. Addressing these challenges will require collaborative efforts between imaging scientists, oncologists, and regulatory bodies.

### 6.2. Future Perspectives

Future progress in pancreatic cancer molecular imaging is anticipated to be driven by several key avenues.

The integration of artificial intelligence (AI) with molecular imaging in PC represents a transformative advance in the early detection and management of this highly aggressive malignancy. Recent studies highlight the potential of AI to revolutionize the diagnostic landscape by enhancing the sensitivity and specificity of imaging modalities, thereby facilitating earlier detection and improved prognostication [220,221]. Beyond early detection, this integration also enables the discovery of prognostic and predictive biomarkers essential for personalized treatment planning. The convergence of multiplexed immunosensors, nanotechnology, and AI-driven data integration is emerging as a groundbreaking framework for early diagnosis of pancreatic cancer [222]. Furthermore, a multimodal AI fusion model that combines deep learning-based imaging analysis with machine learning-based clinical predictions has been shown to significantly improve diagnostic accuracy, sensitivity, and specificity in pancreatic cancer detection [223]. Collectively, these AI technologies offer a robust decision-support tool that can facilitate earlier diagnosis and optimize clinical decision-making, ultimately improving patient outcomes.

Multimodal imaging has become a pivotal strategy in molecular targeted imaging of pancreatic cancer, offering complementary advantages that enhance both diagnostic precision and therapeutic efficacy. Given the disease’s complex nature and poor prognosis, integrating multiple imaging modalities enables a more comprehensive assessment. For example, tumor-specific multimodality approaches combining photoacoustic and fluorescence optical imaging have been shown to improve intraoperative detection of pancreatic cancer significantly [224]. Similarly, multifunctional theranostic platforms—such as organosilica nanomedicine integrating fluorescence, MRI, and photothermal imaging—further demonstrate the potential of multimodal imaging to improve diagnosis and treatment [178]. As novel imaging agents and platforms continue to emerge, multimodal imaging is poised to further advance the management of pancreatic cancer.

Enhancing nanoplatform penetration through the dense stromal barrier represents a critical frontier in pancreatic cancer research. The PDAC tumor microenvironment—characterized by dense desmoplastic stroma, high interstitial pressure, and immunosuppressive features—necessitates innovative nanoplatform designs that enable active targeting, size switching, or enzyme-mediated penetration [225]. Promising strategies to overcome these physical barriers include tumor-penetrating peptides, macrophage-mediated delivery, and extracellular matrix-degrading components, as well as nanocarrier systems tailored to address the desmoplastic and immunosuppressive microenvironment [226]. Beyond structural optimization, stimuli-responsive nanoparticle platforms offer an additional dimension of functionality. The integration of artificial intelligence into the design of these systems has further accelerated their optimization, enabling the rapid identification of structure–function relationships and prediction of tumor-specific accumulation [227]. Recent work by Chow et al. [228] exemplifies the transformative potential of integrating nanomaterial-based molecular imaging with AI. This framework not only refines the quantitative accuracy and targeting specificity of multimodal imaging (including MRI, CT, PET, and PAI), but also pioneers real-time dosimetric prediction and individualized contrast protocol optimization.

Novel targets for early detection represent a critical unmet need in pancreatic cancer imaging. Current probes primarily target advanced-stage disease biomarkers, with limited capability for detecting precursor lesions such as PanIN or IPMN [229]. Emerging targets associated with early oncogenic transformation, including mutant KRAS protein, altered glycosylation patterns, and specific microRNA signatures, offer exciting opportunities to develop next-generation imaging agents that can detect malignancy at its inception.

Personalized radionuclide therapy and dosimetry-guided treatment represent a rapidly maturing theranostic paradigm. The combination of diagnostic imaging with targeted radionuclide therapy using matched targeting ligands and therapeutic isotopes enables individualized treatment planning based on precise dosimetric calculations. As regulatory frameworks evolve and clinical evidence accumulates, theranostics is expected to transition from specialized centers to broader clinical practice.

The effective clinical application of molecular imaging in pancreatic cancer relies on standardized imaging protocols, unified quantitative metrics, and collaborative networks across multiple institutions. International consensus guidelines for image acquisition, analysis, and reporting, similar to those developed for PSMA imaging in prostate cancer. It will be essential for enabling large-scale clinical trials and facilitating regulatory approval. With advancements in synthetic chemistry, protein engineering, and imaging instrumentation, molecular imaging is anticipated to transition from a visualization tool to a key decision-making technology in precision medicine for pancreatic cancer.

## 7. Conclusions

Molecular imaging is fundamentally transforming the precision oncology landscape for pancreatic cancer (PC) by facilitating earlier detection, refining staging accuracy, predicting therapeutic efficacy, and enabling real-time intraoperative navigation. A diverse repertoire of targeted probes—directed against biomarkers such as FAP, integrins, PD-L1, Trop2, EGFR, and c-Met—is effectively surmounting the sensitivity and specificity constraints inherent to conventional imaging modalities. Consequently, these advancements enhance the delineation of micrometastatic disease, characterize intratumoral heterogeneity, and inform tailored therapeutic strategies. These imaging platforms, encompassing monoclonal antibodies, nanobodies, affibodies, peptides, small molecules, and nanoparticle-based agents, have demonstrated robust target affinity, superior contrast-to-noise ratios, and favorable safety profiles, thereby establishing a critical foundation for the implementation of personalized medicine in PC management.

Nevertheless, the broad clinical translation of molecular imaging in PC remains constrained by the tumor’s unique biological barriers—specifically, a dense desmoplastic stroma, a profoundly immunosuppressive microenvironment, and marked intratumoral heterogeneity. These challenges are compounded by technical and regulatory hurdles about probe pharmacokinetics, protocol standardization, scalable manufacturing, and comprehensive long-term biosafety evaluation. Emerging innovations in multimodal hybrid imaging, rational design of dual-targeting ligands, AI-driven image analytics, and integrated theranostic platforms hold significant promise for elevating molecular imaging toward more robust and clinically actionable roles. Through multidisciplinary collaboration, these advancements are set to significantly improve diagnostic accuracy, treatment effectiveness, patient survival, and quality of life.

## Figures and Tables

**Table 1:** Overview of imaging probe classes for molecular imaging.

Probe Class	Representative Examples	Molecular Weight	Tumor Penetration	Clearance Half-Life	Imaging Window	Tumor-to-Background Ratio (TBR)
Full-length antibody	Trastuzumab, Cetuximab, Zolbetuximab	~150 kDa	Average	18–21 days (IgG1/2/4) ~7 days (IgG3)	3–7 days	high TBR after extended clearance periods
Nanobody /affibody	Anti-CD47 VHH, Anti-HER2 affibody	~12–15 kDa	Good	0.5–2 h	1–4 h	high TBR at early time points
Peptide	RGD, FAPI, NTSR1-targeting peptides	~1–5 kDa	Excellent	20–60 min	2–24 h	target-dependent TBR
Small molecule	FAPI-04, FAPI-46, AZD9574	<1 kDa	Excellent	0.5–2 h	0.5–4 h	Moderate TBR, fast distribution but limited specificity
Nanoparticle platform	cRGD-CPNP, SiO2@DOX, Au@MSN	~10–200 nm	Good	Variable (hours-days)	Variable (hours-days)	Moderate TBR mediated by EPR effect

**Table 2:** Overview of molecular imaging probes for pancreatic cancer.

Target	Year of Publication	Author	Molecular Imaging	Probe	Probe Class
FAP	2025	Wang et al. [159]	immunoPET	[^18^F]F-AlF-NOTA-FAPI-04	small molecule
	2025	Li et al. [160]	immunoPET	[^18^F]F-FAPI-04	small molecule
	2025	Qiu et al. [161]	immunoPET	[^68^Ga]Ga-FAPI-04	small molecule
	2025	McGahan et al. [162]	immunoPET	[^68^Ga]Ga-FAPI	small molecule
	2025	Zhang et al. [163]	PET/MRI	[^68^Ga]Ga-DOTA-FAPI-04	small molecule
	2024	Liu et al. [164]	SPECT	[^131^I]I-FAP-2286	polypeptide
PDGFRβ	2025	Li et al. [16]	immunoPET	[^64^Cu]Cu-NOTA-ZPDGFRβ	polypeptide
TGFβ	2023	Li et al. [165]	immunoPET	[^68^Ga]Ga-P144	polypeptide
Collagen Type I	2024	Esfahani et al. [18]	PET/MRI	[^68^Ga]Ga-CBP8	polypeptide
EDB-FN	2024	Zhang et al. [166]	MRI, fluorescence imaging	ZD2-Gd-DOTA-Cy7	polypeptide
Integrin αvβ6	2025	Zhang et al. [167]	immunoPET	[^18^F]AlF-Glc-αvβ6L, [^18^F]AlF-Asp2-αvβ6L	polypeptide
	2025	Pang et al. [168]	immunoPET	[^68^Ga]Ga-αvβ6-2	polypeptide
	2023	Ganguly et al. [169]	immunoPET	[^68^Ga]Ga-DOTA-5G, [^68^Ga]Ga-DOTA-ABM-5G	polypeptide
	2022	Nakamoto et al. [170]	immunoPET	[^18^F]-FP-R01-MG-F2	polypeptide
	2021	Sachindra et al. [171]	SPECT	[^177^Lu]Lu-DOTA-integrin αvβ6 knottin	polypeptide
Integrin α5	2021	Wang et al. [172]	SPECT	[^124^I]I-AV3	polypeptide
Integrin α6	2023	Chen et al. [173]	immunoPET	[^18^F]AlF-NOTA-RD2	small molecule
Integrin αvβ3	2024	Chen et al. [174]	NIR, PAI	cRGD-CPNPs	polypeptide
	2021	Jin et al. [175]	SPECT	[^99m^Tc]Tc-3PRGD_2_	polypeptide
Integrin αvβ3 EGFR	2023	Li et al. [176]	immunoPET	[^64^Cu]Cu-NOTA-RGD, [^64^Cu]Cu-NOTA-GE11, [^64^Cu]Cu-NOTA-RGD-GE11	polypeptide
PD-L1	2023	Xiang et al. [177]	immunoPET	[^68^Ga]Ga-HBED-CC-WL-12	polypeptide
	2024	Zhang et al. [178]	MRI, NIR	DCCGP	nanoparticle
MUC17 CD3	2025	Wen et al. [179]	immunoPET	[^89^Zr]Zr-M17C3	antibody derivative
CD47	2023	Liang et al. [180]	immunoPET	[^68^Ga]Ga-NOTA-C2, [^89^Zr]Zr-DFO-ABDC2	nanobody
CD44	2025	Tang et al. [181]	immunoPET	[^89^Zr]Zr-1M2E3	mAb
EGFR VEGF	2023	Wang et al. [182]	PAI, BLI	Bi-fp50	polypeptide
Trop2	2022	Chen et al. [183]	immunoPET	[^89^Zr]Zr-DFO-AF650	mAb
	2022	Li et al. [184]	immunoPET, SPECT, RIT	[^64^Cu]Cu-NOTA-hIMB1636, [^177^Lu]Lu-DOTA-hIMB1636	mAb
EGFR	2021	Matsumoto et al. [185]	immunoPET	[^64^Cu]Cu-NCAB001	mAb
	2021	Matsumoto et al. [186]	immunoPET	[^64^Cu]Cu-NCAB001	mAb
MUC5AC	2021	Henry et al. [187]	immunoPET	[^89^Zr]Zr-DFO-RA96	mAb
	2022	Nakata et al. [188]	immunoPET	[^89^Zr]Zr-hNd2, [^225^Ac]Ac-hNd2	mAb
MUC4	2025	Jaiswal et al. [189]	NIRF	anti-MUC4-IR800	mAb
ACE2	2024	Zhou et al. [190]	SPECT	[^99m^Tc]Tc-cyc-DX600	polypeptide
EphA2	2025	Sharma et al. [191]	immunoPET	[^68^Ga]Ga-AJ201	polypeptide
c-Met	2025	Sun et al. [192]	immunoPET	[^68^Ga]Ga-NOTA-PFCM01	nanobody
CSPG4	2025	Wang et al. [193]	immunoPET	[^68^Ga]Ga-DOTA-LS10, [^68^Ga]Ga-DOTA-SH11, [^68^Ga]Ga-DOTA-TH10	polypeptide
Hsp90	2022	Wang et al. [194]	immunoPET	[^18^F]F-NOTA-Dimer-San A	polypeptide
MT1-MMP	2025	Yang et al. [195]	immunoPET	[^68^Ga]Ga-DOTA-BT1718	small molecule
ST14	2025	Peng et al. [196]	immunoPET	[^68^Ga]Ga-ST14-06	small molecule
5T4	2025	Kong et al. [197]	immunoPET	[^18^F]AlF-RESCA-H006	sdAb
PARP-1	2025	Gao et al. [198]	SPECT	[^68^Ga]Ga-DOTA-PEG-PARPi, [^177^Lu]Lu-DOTA-PEG-PARPi	small molecule
	2025	Patel et al. [199]	immunoPET	[^18^F]AZD9574	small molecule
uPAR	2025	Luo et al. [200]	MRI, NIR	uPAR-ICG-FVIOs	mAb
	2024	Pratt et al. [201]	immunoPET	[^89^Zr]Zr-DFO-anti-huPAR, [^89^Zr]Zr-DFO-anti-muPAR	mAb
H-type-3	2023	Kuroda et al. [202]	immunoPET	[^18^F]FB-rBC2LCN	small protein
KRAS	2024	Liu et al. [203]	immunoPET	(2S,4R)-4-[^18^F]FGln	small molecule
NTSR1	2025	Potemkin et al. [204]	SPECT	[^99m^Tc]Tc-HYNIC-NTSR1 antagonist, [^99m^Tc]Tc-tricarbonyl-NTSR1 antagonist	small molecule
GRPR	2023	Tu et al. [205]	SPECT, NIRF	[^99m^Tc]Tc-HYNIC-PEG4-GB-6, MPA-PEG4-GB-6	polypeptide
NPC1L1	2025	Dong et al. [206]	CEUS, NIRF	CHOL@GEM-NBs	nanoscale synthetic nanocarrier
CA199	2023	Chen et al. [207]	SPECT	[^177^Lu]Lu-TE-1132	polypeptide
	2025	Cox et al. [208]	NIRF	CA19-9–IRDye800CW	mAb
CEA	2023	van Manen et al. [209]	NIRF	NbCEA5-ZW800, NbCEA5-ZW800-1	nanobody
TLR2	2024	Huynh et al. [210]	NIRF	TLR2L-800	small molecule
MCTs	2025	Shi et al. [211]	immunoPET	[^18^F]FEtO-CHC	small molecule
FR	2021	Lee et al. [212]	immunoPET, SPECT, BLI	[^124^I]HIB-L1, [^124^I]IPH-L2, [^124^I]HIB-ether-L3, [^124^I]HIB-L4	nanoparticle
DPP-IV/IX	2021	Takahashi et al. [213]	fluorescence imaging	GP-HMRG	small molecule
Nucleus	2025	Su et al. [214]	SPECT	[^125^I]I-TiO_2_-TAT/HA_2_	polypeptide
NTR	2025	Hu et al. [215]	NIRF, PAI	SiRho-SHD-NTR	small molecule
pH/GSH Dual-Responsive	2025	Zhang et al. [17]	MRI	Au@H-MP NMs	nanoparticle
Cys/GSH Responsive	2024	Li et al. [15]	NIRF, PAI	HYD-BX, HYD-DX	small molecule
BSA/AND/Glut	2025	Amaolo et al. [216]	MRI	Gd-PLGA NPs	nanoparticle

*ACE2*, Angiotensin-Converting Enzyme 2; *BiTE*, Bispecific T-cell Engager; *BLI*, Bioluminescence Imaging; *c-Met*, Mesenchymal–Epithelial Transition factor; *CA19-9*, Carbohydrate Antigen 19-9; *CAF*, Cancer-Associated Fibroblast; *CEA*, Carcinoembryonic Antigen; *CEUS*, Contrast-Enhanced Ultrasound; *CSPG4*, Chondroitin Sulfate Proteoglycan 4; *CT*, Computed Tomography; *ECM*, Extracellular Matrix; *EDB-FN*, Extra Domain B Fibronectin; *EGFR*, Epidermal Growth Factor Receptor; *EphA2*, Erythropoietin-Producing Hepatocellular Receptor A2; *FAP*, Fibroblast Activation Protein; *FR*, Folate Receptor; *GRPR*, Gastrin-Releasing Peptide Receptor; *GSH*, Glutathione; *Hsp90*, Heat Shock Protein 90; *IPMN*, Intraductal Papillary Mucinous Neoplasm; *KRAS*, Kirsten Rat Sarcoma viral oncogene; *MCT*, Monocarboxylate Transporter; *MRI*, Magnetic Resonance Imaging; *MT1-MMP*, Membrane-Type 1 Matrix Metalloproteinase; *NIRF*, Near-Infrared Fluorescence; *NPC1L1*, Niemann–Pick C1-Like 1; *NTSR1*, Neurotensin Receptor 1; *PAI*, Photoacoustic Imaging; *PanIN*, Pancreatic Intraepithelial Neoplasia; *PARP-1*, Poly(ADP-ribose) Polymerase 1; *PD-L1*, Programmed Death-Ligand 1; *PDAC*, Pancreatic Ductal Adenocarcinoma; *PDGFRβ*, Platelet-Derived Growth Factor Receptor beta; *PET*, Positron Emission Tomography; *RGD*, Arginine–Glycine–Aspartic acid; *SNR*, Signal-to-Noise Ratio; *SPECT*, Single Photon Emission Computed Tomography; *SUV*, Standardized Uptake Value; *TGFβ*, Transforming Growth Factor beta; *TLR2*, Toll-Like Receptor 2; *Trop2*, Trophoblast Cell-Surface Antigen 2; *uPAR*, Urokinase Plasminogen Activator Receptor.

## Data Availability

No new data were created or analyzed in this study. Data sharing is not applicable to this article.
